# Determining the Phylogenetic and Phylogeographic Origin of Highly Pathogenic Avian Influenza (H7N3) in Mexico

**DOI:** 10.1371/journal.pone.0107330

**Published:** 2014-09-16

**Authors:** Lu Lu, Samantha J. Lycett, Andrew J. Leigh Brown

**Affiliations:** 1 Institute of Evolutionary Biology, University of Edinburgh, Ashworth Laboratories, Edinburgh, United Kingdom; 2 University of Glasgow, Institute of Biodiversity, Animal Health and Comparative Medicine, Glasgow, United Kingdom; Icahn School of Medicine at Mount Sinai, United States of America

## Abstract

Highly pathogenic (HP) avian influenza virus (AIV) H7N3 outbreaks occurred 3 times in the Americas in the past 10 years and caused severe economic loss in the affected regions. In June/July 2012, new HP H7N3 outbreaks occurred at commercial farms in Jalisco, Mexico. Outbreaks continued to be identified in neighbouring states in Mexico till August 2013. To explore the origin of this outbreak, time resolved phylogenetic trees were generated from the eight segments of full-length AIV sequences in North America using BEAST. Location, subtype, avian host species and pathogenicity were modelled as discrete traits upon the trees using continuous time Markov chains. A further joint analysis among segments was performed using a hierarchical phylogenetic model (HPM) which allowed trait rates (location, subtype, host species) to be jointly inferred across different segments. The complete spatial diffusion process was visualised through virtual globe software. Our result indicated the Mexico HP H7N3 originated from the large North America low pathogenicity AIV pool through complicated reassortment events. Different segments were contributed by wild waterfowl from different N. American flyways. Five of the eight segments (HA, NA, NP, M, NS) were introduced from wild birds migrating along the central North American flyway, and PB2, PB1 and PA were introduced via the western North American flyway. These results highlight a potential role for Mexico as a hotspot of virus reassortment as it is where wild birds from different migration routes mix during the winter.

## Background

Migratory birds are major candidates for long-distance dispersal of zoonotic pathogens and low pathogenicity (LP), avian-origin influenza A viruses (AIVs) are widely distributed in free-ranging water birds [1]. Wild birds spread their viruses to other wild as well as domestic birds as they migrate through an area, allowing extensive reassortment [2]. Once introduced into poultry (especially chickens and turkeys), LPAI may switch to high pathogenic viruses (HPAI) with the introduction of basic amino acid residues into the haemagglutinin cleavage site, which is associated with a high mortality rate in poultry [3], [4]. We have recently shown that a higher inter-subtype reassortment rate can be found in wild Anseriformes than domestic Galliformes in the internal segments of Eurasian AIV, indicating the wild bird population was the source of the new reassortants, rather than domestic poultry [5].

Migrating wild birds have been implicated in the spread and emergence of HPAI such as HP H5N1 and H7N3. Viral transmission between wild birds and domestic poultry, and consequent genetic exchange, has contributed to genomic reassortment which confounded disease control efforts [6], [7]. Although predictors of such outbreaks have long been sought, surveillance in wild birds in North America has failed to provide a clear early warning signal. Three H7N3 HPAI events in poultry have occurred in North Americas since 2000, and, in one case, it was reported that the outbreak H7N3 AIV were transmitted from poultry to humans [8]. Phylogenetic analyses indicated that each of these H7N3 HPAI strains had a close relationship with LPAI isolated from wild birds sampled in neighbouring provinces [9], [10], [11].

In June 2012, H7N3 HPAI outbreaks were found in poultry farms in Jalisco state in Mexico, a region of high poultry density [12] and concurrent infections of humans with this HPAI A (H7N3) virus (2 cases) have been confirmed [13]. The outbreak has been affecting broilers, breeders, layers and backyard poultry in the Mexican States of Jalisco, Aguascalientes, Guanajuato and Puebla: the latest outbreak reported by the World Organization for Animal Health (OIE) was on 19^th^ May 2014. Ongoing epidemiological investigations have implicated contact with wild birds as a factor in the outbreaks [12]. However, the specific origin of the novel outbreak strain and its relationship to the previous outbreak strains is not known.

The aim of our study was to investigate the origin of the precursor strain of the Mexico H7N3, using a Bayesian phylogeographic inference framework by reconstructing the spatiotemporal spread of AIV from wild birds in North America.

## Results

### Phylogenetics of the HPAI H7N3 Mexico with north America AIV

**Figure 1:**
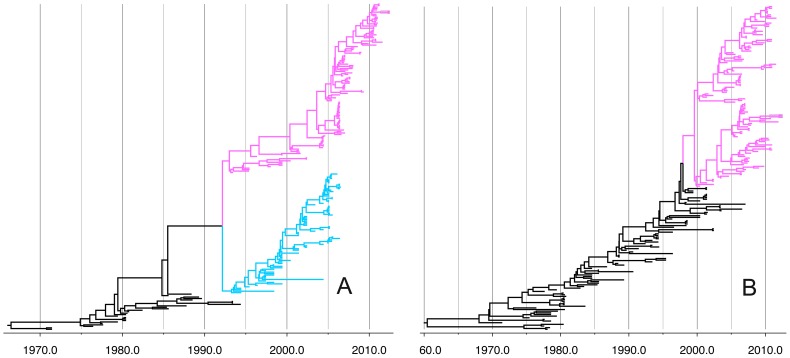
Phylogenies of the H7 and N3 segments of all available North American AIV. A: HA. Sequences in grey are from before 1990; the clade colored blue is composed of H7N2 AIV isolated from a single surveillance in poultry in New York; the clade colored pink was selected for time-scaled phylogenetic analysis. B: NA tree. The uncoloured sequences are from before 2000; the AIV clade in pink was selected for time-scaled phylogenetic analysis.

To investigate the origin of the AIV causing the HPAI H7N3 outbreak in Mexico in 2012, an initial phylogenetic analysis using Maximum likelihood was performed for each segment of both the outbreak sequences and a background dataset which comprised all available AIV of North American AIV lineages (Figure 1). The phylogenetic trees of all available H7 segments in north American showed that AIV isolated in recent years have diverged from those before 1990 (Figure 1A). In addition, in the HA segment a sub-lineage mainly composed of H7N2 AIV from domestic birds in New York state is clearly separate from the recent lineage composed of AIV from wild birds, which indicates extensive diversity of LP AIV in wild and domestic birds. Since 2000, the N3 NA segment of North American AIV has split into two separate lineages (Figure 1B). The mechanism for maintenance of this divergence remains unknown as viruses from both lineages co-circulate in geographically overlapping host populations, mainly wild waterfowl.

Diverse reassortment events involving the six internal segments can be inferred from the maximum likelihood phylogenies of 2343 North American AIV. Clades identified in the phylogeny for one segment (e.g., PB2) are not maintained in the phylogenies of other internal segments (Figure S1). In addition, internal segments of AIV viruses isolated in distant locations can be closely related to each other within the same time period, which suggests not just frequent reassortment but also rapid movement of influenza viruses across North America.

Sequences for time-scaled phylogenetic analysis were selected from the closest clades to the novel H7N3 HPAI viruses on the maximum likelihood tree of each segment. This dataset comprised 427 AIV strains collected over a 12 year period (2001 to 2012). The time to the most recent common ancestor (TMRCA) for each segment of the novel HPAI H7N3 in Mexico was estimated from the time-scaled phylogenetic trees. The HPAI H7N3 strains sampled in Mexico shared similar common ancestors among different genes between October 2011 and March 2012, i.e. during the winter of 2011–2012 (Table 1). The common ancestor of the HPAI H7N3 Mexico outbreak and the closest related avian influenza strains existed between 1.1 to 3.9 years ago, which varied among their different genomic segments (Table 1). The difference in unsampled diversity among gene segments suggested that the reassortment of North American AIV lineages which led to the H7N3 Mexico outbreak may have involved several events spread over this time period. This can be seen by comparing the closest related strains in the phylogenetic tree for any segment: they can be quite distant from the H7N3 Mexico strain in the other segments. This result supports our hypothesis of the occurrence of multiple reassortment events.

**Table 1:** Time of the most recent common ancestors for the Mexico H7N3 virus.

Gene	TMRCA^a^	Most closely related strain^b^
HA	20/3/2012 (29/11/2011, 23/5/2012)	A/north shoveler/Missouri/2010 (H7N3)
NA	4/1/2012 (10/8/2011, 23/4/2012)	A/American green-winged teal/Illinois/2010 (H2N3)
PB2	30/10/2011 (8/6/2011, 25/2/2012)	A/mallard/California/198/2012 (H11N9)
PB1	10/10/2011 (10/10/2010, 28/1/2012)	A/American green-winged teal/California/123/2012 (H1N1)
PA	18/11/2011 (3/7/2011, 26/3/2012)	A/American green-winged teal/California/123/2012 (H1N1)
NP	21/1/2012 (16/8/2011, 5/5/2012)	A/American green-winged teal/Mississippi/2012 (H11N9)
M	2/11/2011 (7/0/2011, 2/5/2012)	A/American green-winged teal/Illinois/2008 (H10N7)
NS	25/10/2011 (25/3/2011, 17/1/2012)	A/American green-winged teal/Illinois/2010 (H10N7)

a Time of the most recent common ancestors (TMRCA) of each segment of the novel Mexico H7N3 virus are represented in the order of date/month/year. The values in parentheses represent the 95% HPD intervals.

b The strains are identified are those most closely related to the outbreak strains in each tree phylogeny in this study.

**Figure 2:**
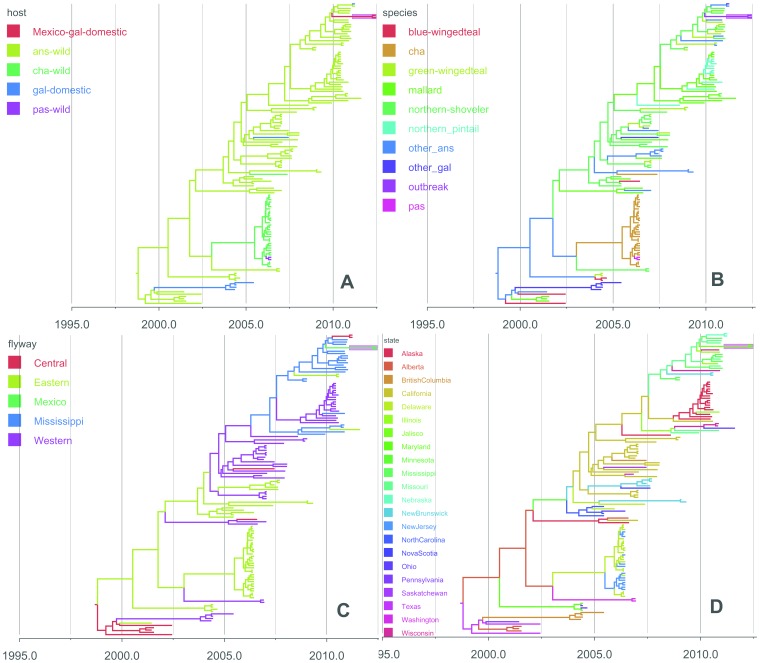
Maximum clade credibility (MCC) phylogenies for the HA segment. Branches are coloured according to the 4 discrete traits (host order, host species, flyway and location) on internal nodes. Mexican outbreak strains are highlighted with pink. A: Host order. Five host orders are labelled on HA tree: wild birds of the order Anseriformes (ans-wild); wild birds of the order Charadriiformes (cha-wild); wild birds of the order Passeriformes (pas-wild); domestic birds of the order Galliformes and Mexico H7N3 outbreak in the order Galliformes (gal-domestic-Mexico). B: Host species. Wild Anseriformes are classified into the five main species and a group comprising the other rarer species of Anseriformes in this study: mallard (Anas platyrhynchos), northern pintail (Anasacuta), northern shoveller, blue-winged teal, green-winged teal and other Anseriformes (other ans); The order Galliformes are shown as “outbreak” (the H7N3 Mexico outbreak) and “other_gal”; The other orders are shown as: Charadriiformes (cha) and Galliformes (gal), Gruiformes (gru) and Passeriformes (pas). C: Flyway. Four specific North American flyways are labelled on the HA tree: the Atlantic, Mississippi, Central, and Pacific. D: State. 22 states and provinces of the viral sample locations are labelled on the HA tree. The original MCC tree files with all taxa names are deposited in Dryad (doi:10.5061/dryad.j5bf8), and trees for the other 7 segments without taxa names can be found in Figure S2, S3, S4, S5, S6, S7, S8.

Co-circulation of multiple H7 clades was observed in HA across North America. Interestingly, the HPAI H7N3 Mexico strains are not related in HA to the HP H7N3 outbreak in British Columbia in 2004 and 2007, but instead are closely related to a subgroup of H7 AIV (H7N3, H7N8 and H7N9) from wild waterfowl isolated from Nebraska, Illinois, Missouri and Mississippi in 2010 and 2011. The mean estimate of the date of the common ancestor is February 2010 (Figure 2). On the other hand, the picture in NA is different: the closest related strain to that of the Mexico outbreak is a subtype H2N3 AIV isolated from a green winged teal in Illinois in 2010 (Figure S2).

In contrast, the three polymerase encoding gene segments PB2, PB1 and PA of the Mexico outbreak strain belong to lineages composed mainly of AIV found in wild waterfowl in California from the beginning of 2012 (Figure S3, S4, S5), with segments PB1 and PA having the same most closely related strain: A/American green-winged teal/California/123/2012 (H1N1). The other internal segments of the Mexico H7N3 strains have a different origin. From the Bayesian phylogenetic tree of the NP segment, the closest AIV strain to H7N3 Mexico is an H11N9 strain isolated from Mississippi in 2012 (Table 1). The NP segment of the H7N3 Mexico outbreak strain is also in the same lineage as a small number of H7N7 AIV strains carried by northern teal in Illinois/Missouri in the fall of 2010; these strains belong to the same lineage in the HA segment as well (see above and Figures 2 and S6). The NS segment was derived from an H10N7 AIV which was also circulating in the same region at that time (Figure S8). In the M segment, however, Mexico H7N3 strains are distinct from all currently available AIV in North America, suggesting a surveillance gap (Figure S7).

These results indicate that the HPAI H7N3 virus that caused the outbreaks in Mexico is not related to any of the previous H7N3 HPAI outbreaks in North America, nor related to other AI outbreaks (HP H5N2 outbreaks in Mexico in 1994–1995, LP H7 outbreaks in Canada in 2009) in domestic birds in recent years [14], [15]. In addition, no clear pattern of association among the segments of the Mexico H7N3 strains was observed, indicating multiple segment exchange events occurred among North American influenza strains to give rise to it.

### Gene flow of the precursor of the HPAI H7N3 outbreak in Mexico

To further explore the origin of the Mexico outbreak strain, a joint analysis of discrete trait models was performed to estimate the overall genetic transmission process. In this the phylogenetic tree space was sampled independently for each segment, while the transition pattern was jointly estimated in a single analysis as the diffusion parameters being applied in the discrete trait models were assumed to be the same (see methods). Four major factors including seven specific traits were tested by implementing Bayesian stochastic search variable selection (BSSVS): i) host population of AIV (order/species); ii) geographic location of sampled AIV (bird migration flyways/provinces and states of North America); iii) subtype of AIV and iv) virulence (pathogenicity/cleavage sites). For each trait, the evolving process of the HPAI H7N3 in Mexico and closely related AIV can be seen from the reconstructed time-scaled phylogeny of each segment independently (with exception of pathogenicity and cleavage sites which only applied to the HA segment), with the branches colored by the specific trait according to the ancestor trait in the internal nodes (Figure 2 and Figure S2, S3, S4, S5, S6, S7, S8).

**Figure 3:**
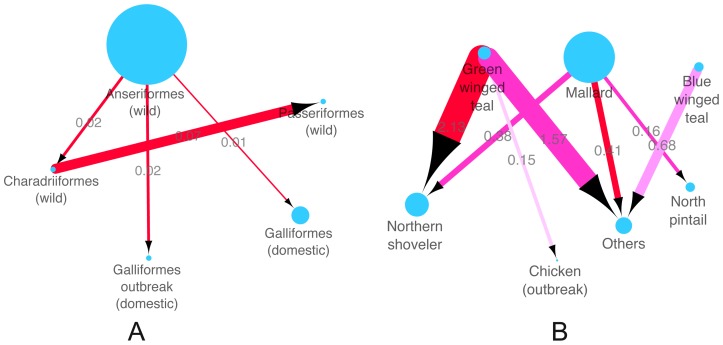
Inferred host transmission networks of Mexican outbreak AIV. A: Host order. Node labels in the nodes are host orders identified following the abbreviations used in the colored phylogenetic trees (Figure 2 and Figures S2, S3, S4, S5, S6, S7, S8): wild birds of the order Anseriformes (ans-wild); wild birds of the order Charadriiformes (cha-wild); wild birds of the order Passeriformes (pas-wild); domestic birds of the order Galliformes and Mexico H7N3 outbreak in the order Galliformes (gal-domestic-Mexico). Arrows show the direction of transmission between two host orders; the arrow weight and the number above each arrow indicates the per capita transmission rate. Node size reflects the number of AIV for each host order (Table S1). Line colours indicate the overall Bayes Factor test support for epidemiological linkage between host orders, Red lines indicate statistical support with BF>100 (very strong support), dark pink lines indicate support with 30<BF<100(strong support), pink lines indicate support with 3<BF<30. B: Host species (Anseriformes only). Wild Anseriformes are further classified into the five main species and a group comprising the other rarer species of Anseriformes in this study: mallard (Anas platyrhynchos), northern pintail (Anasacuta), northern shoveller, blue-winged teal, green-winged teal and other Anseriformes (other ans); As above, arrows show the direction of transmission between two host species; the arrow weight and the number above each arrow indicates the per capita transmission rate. Node size reflects the number of AIV for each host species (Table S3). Line colours indicate the overall Bayes Factor test support for epidemiological linkage between host species, Red lines indicate statistical support with BF>100 (very strong support), dark pink lines indicate support with 30<BF<100(strong support), pink lines indicate support with 3<BF<30.

Host populations of AIV in this study were first analysed by Order: wild Charadriiformes (cha) such as gulls, wild Gruiformes (gru) such as cranes, wild Passeriformes (pas) such as sparrows, domestic Galliformes (gal) such chickens and wild Anseriformes (ans) such as mallards, with Anseriformes comprising the majority of our AIV data set (n = 366/427), see table S1. Among all four strongly supported transitions with Bayes Factor (BF) >100 with mean diffusion rate (R) between 0.01 and 0.07, the highest diffusion rate was found between Charadriiformes and Passeriformes. The other three were found between Anseriformes and other bird orders, and the HPAI H7N3 outbreak in poultry (labelled as Galliformes Mexico) is linked to Anseriformes with strong support (R = 0.02, BF>100) (Figure 3A and Table S5). The results confirm that there has been extensive mixing of influenza A virus between different orders of birds, both wild and domestic.

To explore which host species might have been the direct donor of the Mexico outbreak strains, AIV belonging to Anseriformes were further divided into the five predominant species: mallard (Anas platyrhynchos), northern pintail (Anasacuta), northern shoveller (Anas clypeata), blue-winged teal (Anas discors) and green-winged teal (Anas carolinensis) (Table S2). Species that were sampled at relatively low levels were combined as “other Anseriformes” (Table S2). Multiple statistically supported transitions (with R from 0.15 to 2.13, BF from 6 to over 100) were identified among different host species within this Order, and both mallard and green winged teal are linked to 3 other host species (Figure 3B and Table S6). This analysis indicates the Mexican outbreak strains were most likely to have been transmitted from green winged teals (R = 0.15 and BF = 6).

**Figure 4:**
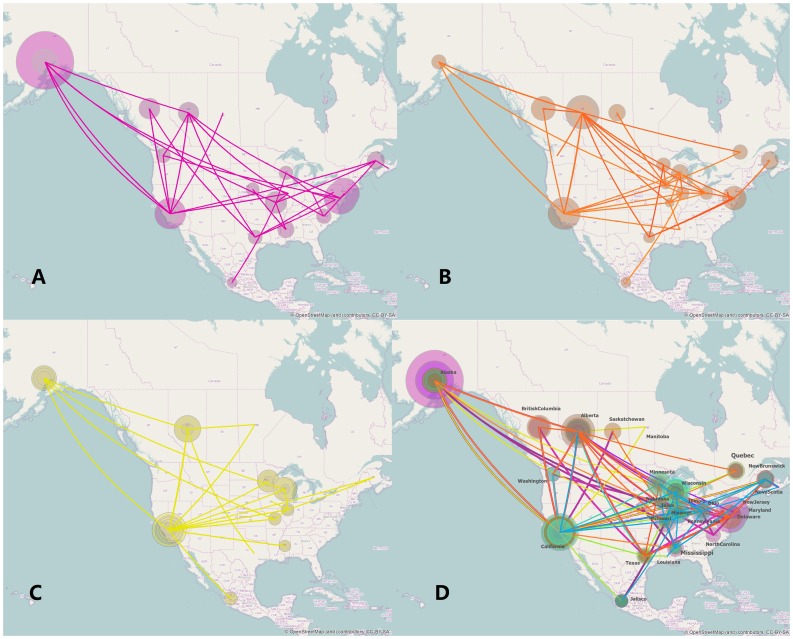
Spatial diffusion of AIV segments of the Mexico outbreak AIV. The first three panels represent three segments separately (A: HA, B: NA, C: PB2) and D represents the spatial transmission of all 8 segments jointly. The plotted lines represent the branches of the MCC trees for different segments, distinguished by color; the size of each circle represents the number of lineages with that location state. The map source for this figure was OpenStreetMap (http://www.openstreetmap.org/). The spatial diffusions of other five segments (PB1, PA NP, M and NS) on the map are shown in Figure S9.

The phylogeographic analysis for each segment of the Mexico HPAI H7N3 strain was summarised by a MCC tree in a geographic context. However, to visualize the evolution process in a spatiotemporal mode we converted the spatial annotated time-scaled phylogeny to an annotated map (Figure 4 and Figure S9). Five segments (HA, NA, NP, M, and NS) of the Mexico HPAI H7N3 strain were introduced directly from different states in central US, while PB2, PB1 and PA were introduced from states in the western region. The introductions of segments from several different geographic locations indicate multiple reassortment events were likely to have been involved in the generation of the novel H7N3 Mexico AIV.

Joint discrete trait analysis of all eight segments indicated frequent gene transfer among locations (states and provinces) where the background AIV sequences were isolated. However, in this initial analysis, no significant support was found between Jalisco (the outbreak state) and any other location, probably due to the large number of possible transitions (26 states, 325 irreversible transition pairs) and the limited number of outbreak strains (3; Table S3). Previous studies have shown that incorporating location greatly improves phylogeographic descriptions of the pattern of virus gene flow [16], [17]. Therefore, we enhanced the statistical power of the analysis in two ways: first by decreasing the number of locations by combining states and provinces into major regions (flyways) and secondly by reducing the number of pairwise transitions possible.

**Figure 5:**
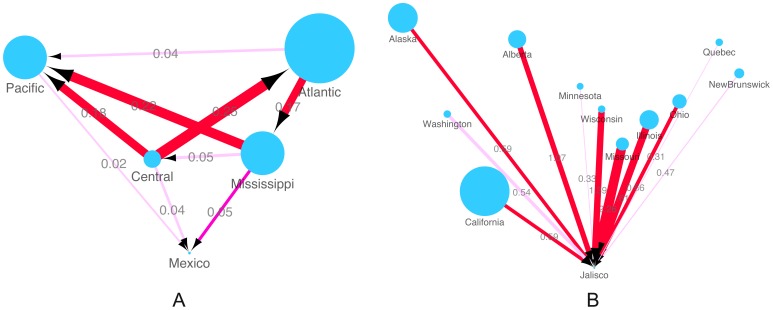
Inferred phylogeographic transmission networks of Mexican outbreak AIV. A: Flyway. AIV transmission among 4 N. American flyways with links to the Mexican outbreak strains. Arrows show the direction of transmission between two flyways; arrow weight and the number above each arrow indicates the per capita transmission rate. Node size reflects the number of AIV for each flyway (Table S6). B: Location. AIV transmission among states/provinces in North America and Jalisco (the Mexican state where the outbreak strains were isolated). Arrows show the direction of transmission between the two states; the arrow weight and the number above each arrow indicates the per capita transmission rate. Node size reflects the number of AIV for each flyway (Table S5).

To aggregate locations we used the known migration routes, or “flyways”: Atlantic, Mississippi, Central, or Pacific (Figure S10). The distributions of avian influenza virus for each flyway are summarized in Table S4, showing a wide range in rate and statistical support (R = 0.02 to 0.25; BF = 3 to >100). Highly significant links [BF>100, Indicator (I)  = 1] were found between major North American flyways, particularly between Atlantic and Mississippi (R = 0.17 exchange/year); Mississippi and Pacific (R = 0.22 exchange/year), Central and Atlantic (R = 0.25 exchange/year) and Central and Pacific (R = 0.18 exchange/year) (Figure 5A and Table S7). Linkages between Atlantic and Pacific flyways, Mississippi and Central flyways were also identified although with weaker support (3<BF<6). The transitions between flyways and the H7N3 HPAI in Mexico are not that strongly supported compared to the between flyway transitions, probably due to the limited number of sequences available from the outbreak AIV, but three direct donors among these flyways to the predecessor of the Mexico outbreak were identified: the Pacific, Central and Mississippi flyways. Among these flows, the transition rate from Mississippi (R = 0.05 exchange/year) is the most strongly supported with BF = 86. In comparison, the link with the Pacific (R = 0.02 exchange/year) and Central (R = 0.04 exchange/year) were weaker (BF = 4; Figure 5B and Table S7). There was no supported link between the Atlantic flyway and Mexico. The results indicated that the HPAI H7N3 in Mexico probably originated from AIV transmitted by wild birds from three different flyways.

Given these findings it appeared likely that the precursor strains were generated somewhere near Mexico as it is the place where birds from the different migration routes meet during winter. To test this hypothesis, we further reduced the number of transitions in the locations transition matrix: transitions between two flyway regions were switched off (by forcing the initial indicator of a given transition pair from 1 to 0, so that this transition pair will not be counted), and those for within flyway transitions and transitions linked to Mexico were maintained. There are 98 non-reversible transition pairs in the new reduced matrix (Table S10). AICM tests (see Methods) revealed that the non-reversible BSSVS model with reduced number of transitions was significantly favoured over the other models with the original matrix (Table 2), indicating the number of transitions has an effect on the performance of discrete trait models. This reduced model has better support than a randomly reduced model with the same number of transition pairs and the same non-reversible BSSVS setting (Table 3); we conclude that gene transitions within flyways and Mexico alone better explain the gene flow of North America AIV than a model incorporating the between flyway transitions.

**Table 2:** AICM estimates for the fit of different discrete trait models.

Joint Discrete trait models	AICM^b^	S.E.^c^	Mod1^d^	Mod2	Mod3	Mod4	Mod5	Mod6	Mod7	Mod8
**Mod1 Reduce_BSSVS_sym** a	**111031**	**+/−7.5**	**−**	**107**	**490**	**712**	**863**	**1102**	**4084**	**6582**
Mod2 Reduce_nonBSSVS_sym	111137	+/−9.449	−107	−	384	605	756	995	3977	6475
Mod3 Reduce_BSSVS_asym	111521	+/−11.798	−490	−384	−	221	372	611	3593	6091
Mod4 Reduce_nonBSSVS_asym	111742	+/−7.822	−712	−605	−221	−	151	390	3372	5870
Mod5 Original_BSSVS_sym	111893	+/−6.668	−863	−756	−372	−151	−	239	3221	5719
Mod6 Original_nonBSSVS_sym	112132	+/−13.455	−1102	−995	−611	−390	−239	−	2982	5480
Mod7 Original_BSSVS_asym	115115	+/−14.662	−4084	−3977	−3593	−3372	−3221	−2982	−	2498
Mod8 Original_nonBSSVS_asym	117612	+/−26.584	−6582	−6475	−6091	−5870	−5719	−5480	−2498	−

a The names of the 8 models (Mod1-8) in the comparison test.

b The estimated AICM score of the posterior: lower values of marginal likelihood indicate a better fit to the data. The model with the best performance is indicated in bold.

c The standard error of the AICM estimated using 1000 bootstrap replicates.

d The AICM comparisons are shown in the matrix composed of columns 4 to 11. In each row of the matrix, the positive value in a cell represents the support for one model (in column 1) over the other (indicated in the column titles). A difference of AICM = 10 is considered to indicate a strong preference for one model over another.

**Table 3:** AICM estimates for the fit of different models with reduced number of transitions.

Joint reduced models^a^	AICM^b^	S.E.^c^	Flyway d	Ran1	Ran2	Ran3	Ran4	Ran5	Ran6	Ran7	Ran8	Ran9	Ran10
**Flyway**	**111031**	**+/−5.2**	**−**	**18**	**70**	**108**	**129**	**157**	**158**	**163**	**171**	**521**	**407822**
Ran1	111049	+/−4.4	−18	−	52	90	111	139	139	145	153	502	407804
Ran2	111101	+/−5.9	−70	−52	−	38	59	87	88	93	101	451	407752
Ran3	111138	+/−14.9	−108	−90	−38	−	21	50	50	55	63	413	407714
Ran4	111160	+/−6.5	−129	−111	−59	−21	−	28	28	34	42	391	407693
Ran5	111188	+/−5.6	−157	−139	−87	−50	−28	−	0	6	13	363	407664
Ran6	111188	+/−4.3	−158	−139	−88	−50	−28	0	−	6	13	363	407664
Ran7	111194	+/−12.8	−163	−145	−93	−55	−34	−6	−6	−	8	357	407659
Ran8	111201	+/−8.6	−171	−153	−101	−63	−42	−13	−13	−8	−	350	407651
Ran9	111551	+/−9.9	−521	−502	−451	−413	−391	−363	−363	−357	−350	−	407301
Ran10	518853	+/−3495.6	−407822	−407804	−407752	−407714	−407693	−407664	−407664	−407659	−407651	−407301	−

a The names of the 11 models (Flyway model and Ran1-10) in the comparison test: Model1 is the non-reversible BSSVS model without between flyway transitions (Flyway); Ran1-10 are models in which the same number of transitions has been switched off, chosen at random (Ran1-10).

b The estimated AICM score of the posterior: lower values of marginal likelihood indicate a better fit to the data. The model with the best performance is indicated in bold.

c The standard error of AICM estimated using 1000 bootstrap replicates.

d Differences of models are shown in the matrix being composed by column 4 to 13. In each row of the matrix, the positive value in a cell represented the support for one model (in column 1) over the other (the column title). A difference of AICM = 10 is considered a strong preference for one model over another.

Considering individual locations, we found 11 locations were linked to the HPAI H7N3 in Mexico, among which 7 showed significantly strong links (BF>100). These were: Alaska (R = 0.59), Alberta (R = 1.07), California (R = 0.59), Illinois (R = 1.61), Missouri (R = 2.26), Ohio (R = 0.66) and Wisconsin (R = 1.39), belonging to the Pacific, Central and Mississippi flyways. AIV from other four states/provinces (Minnesota, New Brunswick, Quebec and Washington) are also linked to Mexico H7N3 AIV but with weaker support (BF = 21 to 30) and lower rate (0.7 to 0.84) (Figure 5A and Table S8). This result indicates the donor locations of the Mexico outbreak are spread widely across North America.

In addition, an extremely complex pattern of linkage between the 52 ancestral subtypes (Table S9) was identified, which confirmed the extent of the reassortment events which had occurred between, especially in the internal segments (full tree with annotation can be found on the Dryad Digital Repository: doi:10.5061/dryad.j5bf8). However, as for locations, no significant linkage between H7N3 in Mexico and other subtypes was identified due to the large number of candidate donor subtypes.

Mexico H7N3 is confirmed in the phylogenetic trees of the HA segment (Figure S11) to have mutated from a LPAI (low pathogenic avian influenza) virus to HPAI after the ancestral virus was introduced into poultry from wild birds (Figure S11A), rather than being associated with previous HP outbreaks. The virulence of HP avian influenza viruses is associated with the appearance of an insertion of multiple basic amino acids at the cleavage site of the HA protein [18]. Categorizing the cleavage sites in this dataset into three types: 1) Insertion, 2) Partial insertion,3) No insertion (Figure S11 C), we find that the H7N3 Mexico strain has a unique insertion - DRKSRHRR - compared to other HPAI strains (Figure S11 C). We conclude the Mexico H7N3 strains originated from a lineage composed of LP strains with partial insertions in the cleavage sites. Similarly, the H7N3 strains which caused an outbreak of HPAI in Canada in 2004 were also derived from LP strains with the same partial insertions, but from a completely separate HA clade, indicating parallel evolution with respect to the acquisition of the multibasic cleavage site, starting from different lineages (Figure S11 B).

## Discussion

We have investigated the origin of the recent highly pathogenic H7N3 outbreaks in Mexico. Our analysis found that the progenitor of the HPAI H7N3 was a reassortant virus with several different origins among the eight segments. We also found that gene segments of AIV in North American wild birds are exchanged at a very high frequency, with no evidence of any restriction which might imply linkage of segments.

Our study confirmed the assumptions of earlier studies based on the HA segment that the outbreak strain derived from wild birds [12], [19], [20]. We have now shown using powerful Bayesian phylogenetic methods that the origin of the HA segments of the Mexico H7N3 strains can be dated to March 2012, and that they fall into a subgroup of H7 AIV (H7N3, H7N8 and H7N9) from wild waterfowl isolated from Nebraska, Illinois, Missouri and Mississippi with a common ancestor around February 2010. We have extended this analysis to all eight segments, thus obtaining the complete evolutionary history of the outbreak avian influenza viruses.

The predecessors of HPAI H7N3 in Mexico were transmitted from migrating waterfowl in North America. Previous cases of periodic transmission of H7N3 viruses from wild birds to gallinaceous poultry in the Americas suggests that these viruses continuously circulate in wild birds, and their propensity to become highly pathogenic after transmission suggests that they have a gene constellation conducive to generating pathogenic variants [21]. H7N3 has been responsible for all lethal influenza outbreaks in poultry in the Americas over the past decade [21]. Experimental studies have also indicated that H7 influenza viruses from the North American lineage have acquired sialic acid-binding properties that more closely resemble those of human influenza viruses and have the potential to spread to naive animals [22]. In parallel, H7 influenza viruses from East Asian migratory waterfowl were introduced into domestic ducks in China on several occasions during the past decade and subsequently reassorted with enzootic H9N2 viruses to generate a novel H7N9 influenza A virus, resulting in 44 human deaths in China (WHO reported in Dec.3^rd^ 2013) since its first detection in March 2013 [23]. These results indicated that AIV of H7 subtypes carried by wild birds are potential threat to mammalian hosts.

Earlier studies showed that shorebirds and gulls in the Americas are more frequently the source of the potential precursors to HP H5 and H7 avian influenza viruses, while in Eurasia, the precursors of HP influenza viruses are usually from duck species [24], [25]. However, we found that wild Anseriformes (ducks and geese) were the origin of the precursor of HPAI H7N3 in Mexico. Anseriformes showed substantial diversity of AIV in North America, and were divided into five avian species groups in our dataset – mallards, northern pintails, northern shovellers, blue-winged teals, green-winged teals, which have specific ranges for breeding, migration and wintering [17]. We found the green winged teal was the species most strongly supported as the direct donor of the predecessor HPAI H7N3 in Mexico. This species nests as far north as Alaska, and migrates along all four flyways. However, the genetic transitions of different segments showed a complicated interaction involving different bird species.

Migrating birds may exchange viruses with other populations at staging, stopover or wintering sites [26]. Many studies have been performed on AIV gene flows in North America during wild bird migration: One such revealed that avian influenza virus exhibits a strongly spatially structured population in North America, and the intra-continental spread of AIV by migratory birds is subject to major ecological barriers, including spatial distance and avian flyway [17]. Earlier studies suggested that AIV exhibits a strongly spatially structured population in North America, with relatively infrequent gene flow among localities and especially between those that are spatially distant or belong to different flyways using phylogeographic analysis [27]. This hypothesis was supported by studies showed that AIV isolates from mallard were linked by migration between sites in central Canada and Maryland but limited reassortment occurred along the inter-migratory flyway routes [28]. However, more recently, the opposite was seen in a another study, which emphasized that the long-term persistence of the influenza A virus gene pool in North American wild birds may be independent of migratory flyways, and the short-term evolutionary consequences of these ecological barriers may be rapidly erased by East-West virus migration [29].

We also found there are genetic interactions between flyways, using a similar discrete trait model. However, to find the strongest link between the Mexican outbreak and potential precursors we found the model had more power when we switched off the between flyway transitions, keeping only the links within flyways and with Mexico. We found that gene flow from three flyways (Pacific, Central and Mississippi) generated the reassortants which acted as the predecessor of HPAI H7N3 in Mexico, and it is possible that the reassortment events occurred in Mexico or farther afield. Flyway boundaries are not sharply defined and both in the northern breeding grounds and the southern wintering grounds there is overlap to some degree. For example, in Panama parts of all four flyways merge into one (http://www.birdnature.com/flyways.html). Birds that are long-distance migrants typically have ranges that extend from the United States and Canada in the summer to Mexico and further south in the winter and nearly all of the migratory birds of the eastern United States, as well as many western species, use the western Mexican Gulf during migration [30].

While the resolution and detection of migration events has been enhanced through increased surveillance in recent years, critical information for wild bird surveillance remains sparse. Only one AIV in Mexico has been published (A/cinnamon teal/Mexico/2817/2006) and it is not related to the new outbreak virus in any of the eight segments (data not shown). We found the origin of the M segment of the H7N3 outbreak gene was distinct from other North American AIV in the dataset, as seen in the phylogenetic trees. In addition, the relatively greater length of branches preceding the outbreak group in other gene segments (Figures 2 and S2, S3, S4, S5, S6, S7, S8) suggests there may be missing intermediates, possibly through insufficient AIV surveillance in Central and South America. Together with previous phylogenetic studies which also mentioned the importance of filling gaps in viral sampling (with record of sampling time and location) in these regions [31], [32], this highlights the need for increased surveillance in those regions.

Overall, by combining the phylogenetic history of AIV with the host distribution and ecology in our analysis, we show the origin of HPAI H7N3 AIV that caused a series of poultry outbreaks in Mexico is an novel reassortant carried by migration of wild waterfowls from different migration flyways in North America throughout the time period studied, and, more importantly, Central America might be a potential hotspot for AIV reassortment events. Our results are useful for identifying the threat of AIV in wild birds and indicate comprehensive surveillance in South and Central America is highly desirable.

## Methods

### Data preparation

The complete genome of three outbreak strains of H7N3 from Mexico (A/chicken/Jalisco/CPA1/2012; A/chicken/Jalisco/12283/2012; A/Mexico/InDRE7218/2012) and all previously published influenza A virus sequences of North American lineage (complete genome only) were downloaded from GenBank on 1st March 2013. Sequences of each gene segment were aligned using MUSCLE v3.5 [33]. Maximum likelihood (ML) phylogenetic trees for each segment were generated using RAxML v7.04 [34], each employing a GTR GAMMA substitution model with 500 bootstraps. We established a full genome dataset which was composed of the same 2343 North American strains for each segment. The HA and NA segments have extremely high divergence between different subtypes, therefore, we used all available H7 and N3 to generate the raw trees of HA and NA segment respectively; While there are diverse reassortment and interaction among the six internal segments, therefore, we constructed the giant ML trees for the internal segments in order to identify the outbreak strains related strains. Background sequences for further study were selected from the closest clades to the novel H7N3 HPAI viruses on the maximum likelihood tree of each segment. The final dataset of 427 AIV strains collected over a 12 year period (2001 to 2012) is displayed in Table S9. In this table the segments selected for analysis for each strain are indicated. For the majority of strains, only one segment is selected (n = 289), while for others more than one segments is included. There are 131 HA (H7) sequences included in the analysis based on their relationship to the Mexico H7N3 strain (1698 nt); other H7 sequences included in the joint analysis are related to the outbreak strain in other segments. For the other segments the distribution is as follows: NA (N3), n = 100 (1410 nt); PB2, n = 86 sequences (alignment length of 2277 nucleotides); PB1, n = 67 (2271 nt); PA, n = 89 (2148 nt); NP, n = 79 (1494 nt); MP, n = 39 (982 nt); and NS, n = 42 (838 nt). The trait information (host order; host species; location; flyway; state; subtype) of these background AIV sequences are also provided in Table S9.

### Time-scaled phylogeny reconstruction

To estimate the origin in time and space of the HPAI H7N3 outbreak strain in Mexico, models in BEAST v.1.7.3 [35], [36] were applied independently to each gene segment (each segment has a different number of AIV sequences). Different combinations of substitution models: general time-reversible (GTR) substitution model+ Γ distributed site-site rate variation [37] and SRD06 [38]; clock models: strict and uncorrelated relaxed lognormal; and population size models: constant size, exponential, skyride models were evaluated by Bayes Factor test. The best fitting model - incorporating a GTR substitution model+ Γ with uncorrelated lognormal relaxed molecular clocks and a constant-population coalescent process prior over the phylogenies was selected. Parameters were estimated using the Bayesian Monte-Carlo Markov Chain (MCMC) approach implemented in BEAST. MCMC chains were run for 100 million states, sampled every 10,000 states with 10% burn-in. MCMC convergence, and effective sample size of parameter estimates were evaluated using Tracer 1.5 (http://beast.bio.ed.ac.uk). Maximum clade credibility (MCC) trees were summarized by using Tree Annotator and visualized by using FigTree v1.4.0 (http://tree.bio.ed.ac.uk/software/figtree/).

A graphical representation of the origin of HPAI H7N3 Mexico was obtained by spatial reconstruction using a Bayesian framework. The SPREAD application [39] was used to convert the estimated divergence times and the spatially-annotated time-scaled phylogeny (by associating each location with a particular latitude and longitude) to a spatiotemporal movement. The mapped objects were exported to keyhole markup language (KML) files and then were visualized by geographic information systems software: ARCGIS (http://www.esri.com/software/arcgis). The map source is OpenStreetMap (http://www.openstreetmap.org/).

### Joint analysis of the transition rates between discrete traits

To reduce the effects of sampling bias and identify reassortment events in each individual segment, a joint analysis of all segments was performed using a hierarchical phylogenetic model (HPM) [36], [40].

In this hierarchical structure the prior on a parameter may be shared between partitioned datasets in order to increase the efficiency of its estimation [41]. This approach has recently been used in an analysis of the phylogeographic history of Dengue virus where information from multiple phylogeographic datasets was combined in a hierarchical setting using an HPM framework [42]. Here, we use HPMs for discrete trait models where each segment is treated as an independent dataset with an individual relaxed clock model and tree prior, but shares the discrete trait model (subtype, location and host) with all other segments resulting in a joint transition matrix for each discrete trait. Note that is it not necessary to have the same taxa in each of the data partitions within this framework, since the transition rate matrix is estimated using the tip patterns and trees from all of the partitions, when there are missing states and transitions in one partition the estimates of those rates comes from the other partitions where the states and transitions are present.

Bayesian stochastic search variable selection (BSSVS) was employed to reduce the number of parameters to those with significantly non-zero transition rates [43]. A Bayes Factor support (BF)>100 was considered to indicate decisive support, whereas 3≤BF≤30, and 30≤BF≤100 indicate substantial, and strong statistical support, respectively. The Bayes Factor threshold was estimated from Rate indicators using the indicator BF tool in the BEAST v1.6.2 package. The default Poisson distribution (with mean equal to the number of states) was used as a prior on the number of rate indicators.

Three factors: host, geographic location and subtype were analysed using discrete trait models in BEAST [44]. Specifically, host population was discretized in terms of bird orders and bird species, while geographic location was discretized in terms of states and migration flyway routes in North America. The hosts of AIV in our study were first categorized into five different bird orders: Anseriformes (ans), Charadriiformes (cha) and Galliformes (gal), Gruiformes (gru) and Passeriformes (pas); The majority of our AIV data set were collected from Anseriformes (366/427), which were further classified as five major species groups: mallard (Anas platyrhynchos), northern pintail (Anasacuta), northern shoveller (Anas clypeata), blue-winged teal (Anas discors), green-winged teal (Anas carolinensis) and other Anseriformes. Each AIV sequence was assigned a discrete geographical state according to its province / state of isolation (in Table S9). In total 26 US states and Canadian provinces were considered in our study. In addition, each state and province was categorised into a specific North American flyway: the Atlantic, Mississippi, Central, or Pacific flyway, following a previous study [17]. Viral sequences were also labelled according to their host species for the analysis of the species contribution to AIV gene flow. The distributions of sequences for each discrete trait (host order, host species, flyway and location) are summarized in Table S1, S2, S3, S4.

These traits were jointly analysed across all eight segments using irreversible substitution models and strict clocks in the discrete trait models. An exponential distribution with mean equal to 1.0 was chosen for the discrete trait clock rate prior in order to favour smaller numbers of transitions across the phylogeny of each segment (especially PB2, M and NS). The parameter values in each trait model were examined using Tracer v1.6. Significant transition rate estimates between discrete traits were calculated using the same methods as in [5] and plotted as a network in Cytoscape v2.8.0 (http://www.cytoscape.org/).

### Flyway-restricted transmission

We further modified the discrete transition model specification in the xml configuration files to reflect the hypothesis the gene flow of AIV carried by migration birds are restricted between flyways but can mix in Mexico (and further south). By default, all possible transitions between states are permitted, but we modified the settings to disallow transitions between locations on different flyways (Table S10). The output from this restricted model can then be compared to the original model. The corresponding indicators for BF thresholds were recalculated based on the reduced number of transitions. Eight discrete trait models were further compared based on 3 settings: 1) with BSSVS or without BSSVS implemented; 2) Asymmetric or symmetric transitions model; 3) with reduced or original matrix (The names of the models are: 1. Reduce_BSSVS_asym; 2. Reduce_nonBSSVS_asym; 3. Reduce_BSSVS_sym; 4. Reduce_nonBSSVS_sym; 5. Original_BSSVS_asym; 6. Original_nonBSSVS_asym; 7. Original_BSSVS_sym; 8. Original_nonBSSVS_sym).

The location trait initially had 26 states, resulting in 325 possible transition rates, however to avoid over-parameterising the model, we reduced the number of transitions in the matrix to 98 to increase the power of the analysis. To demonstrate that it was not the case that the reduced flyway matrix performed best only because of the lower number of parameters, we performed a randomization test with the same number of parameters: we randomly switched off (meaning the indicator of a transition pair was forced from 1 to 0, ensuring that it was not included in the model during sampling) the same number of transitions as were disallowed in the between flyway states matrix. We did this for 10 replicates. We then compared the performance to that of the reduced flyway matrix and we found the latter has the best performance, indicating the model with reduced flyway transition is the best model to explain the spatial transmission with location state.

All models were compared using a posterior simulation-based analogue of Akaike's information reiteration (AICM), by measuring AIC from the posterior of each model in a Bayesian Monte Carlo context, with score >10 as a strong evidence in favour of one model over the others [45], [46]. The comparisons were conducted in Tracer V 1.6 using a further subsampled log file of each model (>1000 states after burn-in). A recently developed model comparison method, estimating marginal likelihoods using stepping stone (SS) sampling [46], [47] was also used to compare the discrete trait models based in order to verify the AICM results. However comparison by SS marginal likelihood estimation was only performed on discrete trait models inferred on the HA segment since the SS method is not yet implemented for the HPM framework. Similar to the joint analysis results by AICM, the SS results also showed the symmetric discrete trait model with reduced matrix in BSSVS is preferred over all the other models with average improvement in marginal likelihood  = 10, and the model with between flyway diffusion pairs switched off was favoured over a randomly reduced matrix with an average improvement in marginal likelihood of 52.

The original tree files from this study are available from the Dryad Digital Repository: doi:10.5061/dryad.j5bf8.

Including:

1. Maximum likelihood (ML) trees of six internal segments PB2, PB1, PA, NP, M and NS for the same 2343 North America AIV strains reconstructed using RAxML v7.04. In each ML tree, the Mexico H7N3 strains are colored in red, and the lineage in which H7N3 Mexico fell is colored in blue;
2. Temporally structured maximum clade credibility (mcc) time-scaled phylogenetic trees of all eight segments were generated using Beast V 1.7.3 and annotated with ancestral state (A: host order; B: host species; C: flyway; D: state/province; E: subtype) recovered from the discrete trait analyses. All the tree files can be visualized in FigTree. V 1.4.0.

## Supporting Information

Figure S1 **Maximum likelihood (ML) phylogenies of the six internal segments of North American AIV.** Phylogenetic trees were generated with the same 2343 strains in each of six internal segments. In the PB2 segment, 86 AIV sequences in the same clade as the H7N3 Mexico strains are labelled in red; these strains are widely scattered in the phylogenetic trees of the other internal segments. The distribution of the colored lineages among different segments can be visualized in the original tree files in Dryad mentioned above.
(TIF)

Figure S2 **Maximum clade credibility (MCC) phylogenies for the NA segment.** Temporally structured maximum clade credibility (mcc) time-scaled phylogenetic tree showing the evolution of NA gene of avian influenza A virus isolated from North American wild birds for each individual gene dataset. Ancestral state (A: host order; B: host species; location; C: flyway; D: state) changes recovered from the discrete trait analyses are indicated by color changes at tree nodes. Mexican outbreak strains are highlighted with pink. A: Host order. Five host orders are labelled on HA tree: wild birds of the order Anseriformes (ans-wild); wild birds of the order Charadriiformes (cha-wild); wild birds of the order Passeriformes (pas-wild); domestic birds of the order Galliformes and Mexico H7N3 outbreak in the order Galliformes (gal-domestic-Mexico). B: Host species. Wild Anseriformes are classified into the five main species and a group comprising the other rarer species of Anseriformes in this study: mallard (Anas platyrhynchos), northern pintail (Anasacuta), northern shoveller, blue-winged teal, green-winged teal and other Anseriformes (other ans); The order Galliformes are shown as “outbreak” (the H7N3 Mexico outbreak) and “other_gal”; The other orders are shown as: Charadriiformes (cha) and Galliformes (gal), Gruiformes (gru) and Passeriformes (pas). C: Flyway. Four specific North American flyways are labelled on HA tree: the Atlantic (Eastern), Mississippi, Central, and Pacific (Western). D: State. 22 states and provinces of the viral sample locations are labelled on HA tree. The same color code also applies to Figures S3, S4, S5, S6, S7, S8.
(TIF)

Figure S3 **MCC phylogenies for the PB2 segment.**
(TIF)

Figure S4 **MCC phylogenies for the PB1 segment.**
(TIF)

Figure S5 **MCC phylogenies for the PA segment.**
(TIF)

Figure S6 **MCC phylogenies for the NP segment.**
(TIF)

Figure S7 **MCC phylogenies for the M segment.**
(TIF)

Figure S8 **MCC phylogenies for the NS segment.**
(TIF)

Figure S9 **Spatial diffusion of AIV segments of the Mexico outbreak.** Panels represent eight segments separately (A: HA, B: NA, C: PB2, D: PB1, E: PA, F: NP, G: M, H: NS) and I represents the spatial transmission of all 8 segments jointly. The Map source for this figure was OpenStreetMap (http://www.openstreetmap.org/). Plotted lines are the branches of the MCC trees of different segments, distinguished by color; the size of each circle represents the number of lineages with that location state.
(TIF)

Figure S10 **North America states and provinces categorized according to flyways.** Provinces and states which belong to Pacific flyways are colored in purple; Central flyways are colored in red; Mississippi flyways are colored in blue; Atlantic flyways are colored in yellow; and Mexico is colored in green. Those colors on the map are the same as the colored branches in the flyway discrete phylogenetic tree in Figure 2 C.
(TIF)

Figure S11 **Virulence evolving on the MCC phylogeny of the HA segment.** HA cleavage site insertions are classified as follows: 1) Insertion: which are multibasic insertions (insertion) which only being found in HP AIV, including HP H7N3 outbreaks strains in Mexico 2012 and in Canada 2004 and 2007; 2) Partial insertion: LP AIV which has a partial insertion of 2 amino acids; 3) No insertion: other LP AIV with no insertion in the cleavage site. (A): Pathogenicity changes (according to high pathogenicity (HP) and low pathogenicity (LP)) are indicated by color changes in the tree; Cleavage site changes (with insertion, partial insertions and no insertions (C) are indicated by color changes at tree nodes.
(TIF)

Table S1 **Host orders distribution of 427 AIV sequences.**
(DOCX)

Table S2 **Host species (Anseriformes only) distribution of 366 AIV sequences.**
(DOCX)

Table S3 **Location (state/province) distribution of 427 AIV sequences.**
(DOCX)

Table S4 **Flyway distribution of 427 AIV sequences.**
(DOCX)

Table S5 **Transmission rates of host orders and Bayes Factor support.**
(DOCX)

Table S6 **Transmission rates of host species and Bayes Factor support.**
(DOCX)

Table S7 **Transmission rates of flyway and Bayes Factor support.**
(DOCX)

Table S8 **Transmission rates of location (state/province) and the Bayes Factor support.**
(DOCX)

Table S9 **AIV sequences being used in this study.** The strain name and subtype, host, location, flyway and the segments being selected in the AIV dataset. Details of the 427 North American AIV sequences used in this analysis. Strain name, subtype, host orders, isolated state/provinces and the correspondent flyways are listed.
(DOCX)

Table S10 **Reduced indicator matrix with between-flyway states turned off.** The indicator matrix of non-reversible BSSVS model contains 325 transition pairs between states, which are all initially switched on (indicator  = 1). In the reduced matrix, pairs of states belonging to different flyways are switched are switch off (indicator  = 0). There are 98 non-reversible transition pairs in the new indicator matrix.
(DOCX)
