# Supplementary figures and images for: Determining the Phylogenetic and Phylogeographic Origin of Highly Pathogenic Avian Influenza (H7N3) in Mexico
Source: PLoS One. 2014 Sep 16;9(9):e107330. doi: 10.1371/journal.pone.0107330 (PMC4165766; doi:10.1371/journal.pone.0107330)

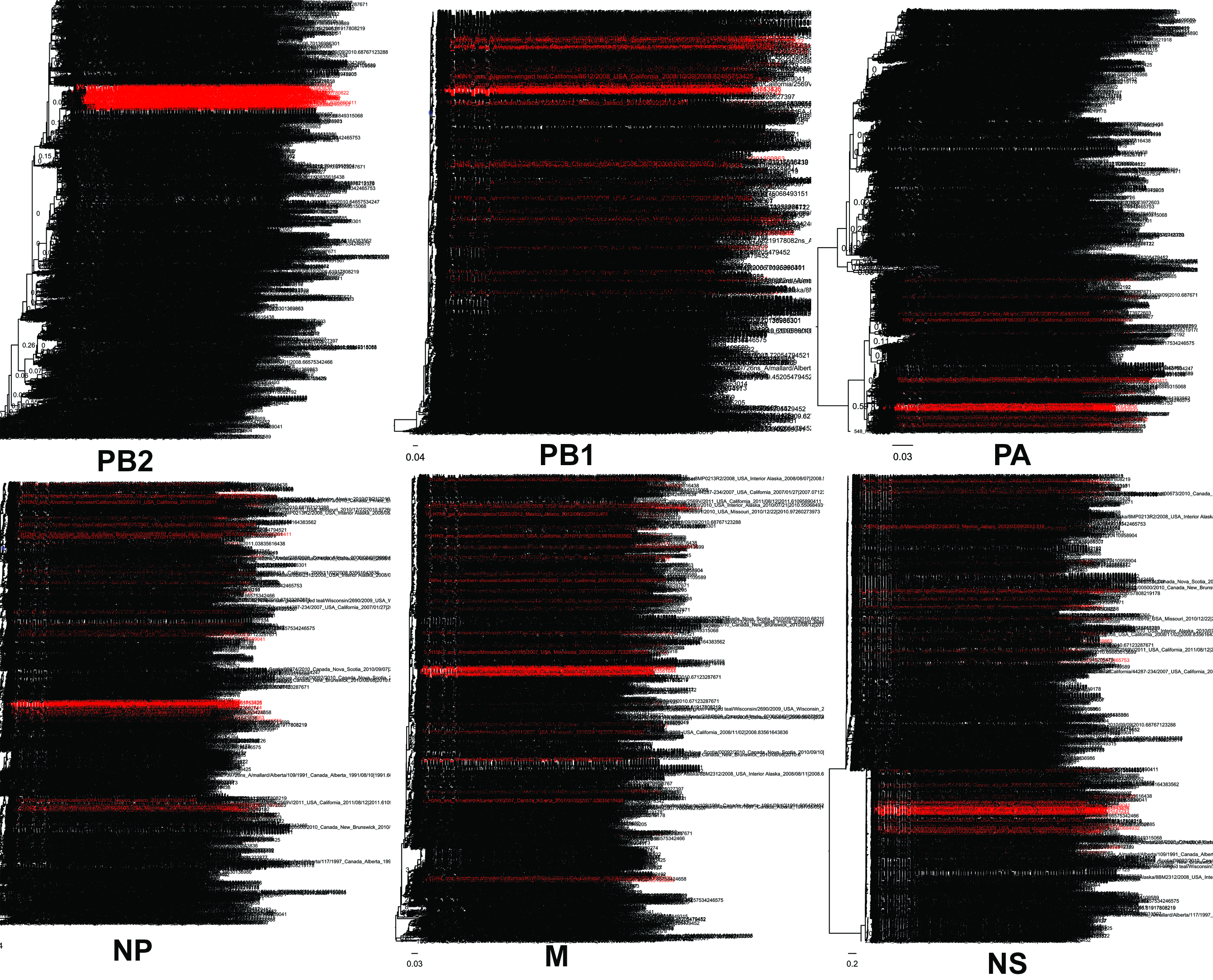

Supplement: Figure S1 — Maximum likelihood (ML) phylogenies of the six internal segments of North American AIV. Phylogenetic trees were generated with the same 2343 strains in each of six internal segments. In the PB2 segment, 86 AIV sequences in the same clade as the H7N3 Mexico strains are labelled in red; these strains are widely scattered in the phylogenetic trees of the other internal segments. The distribution of the colored lineages among different segments can be visualized in the original tree files in Dryad mentioned above. (TIF) [file pone.0107330.s001.tif]

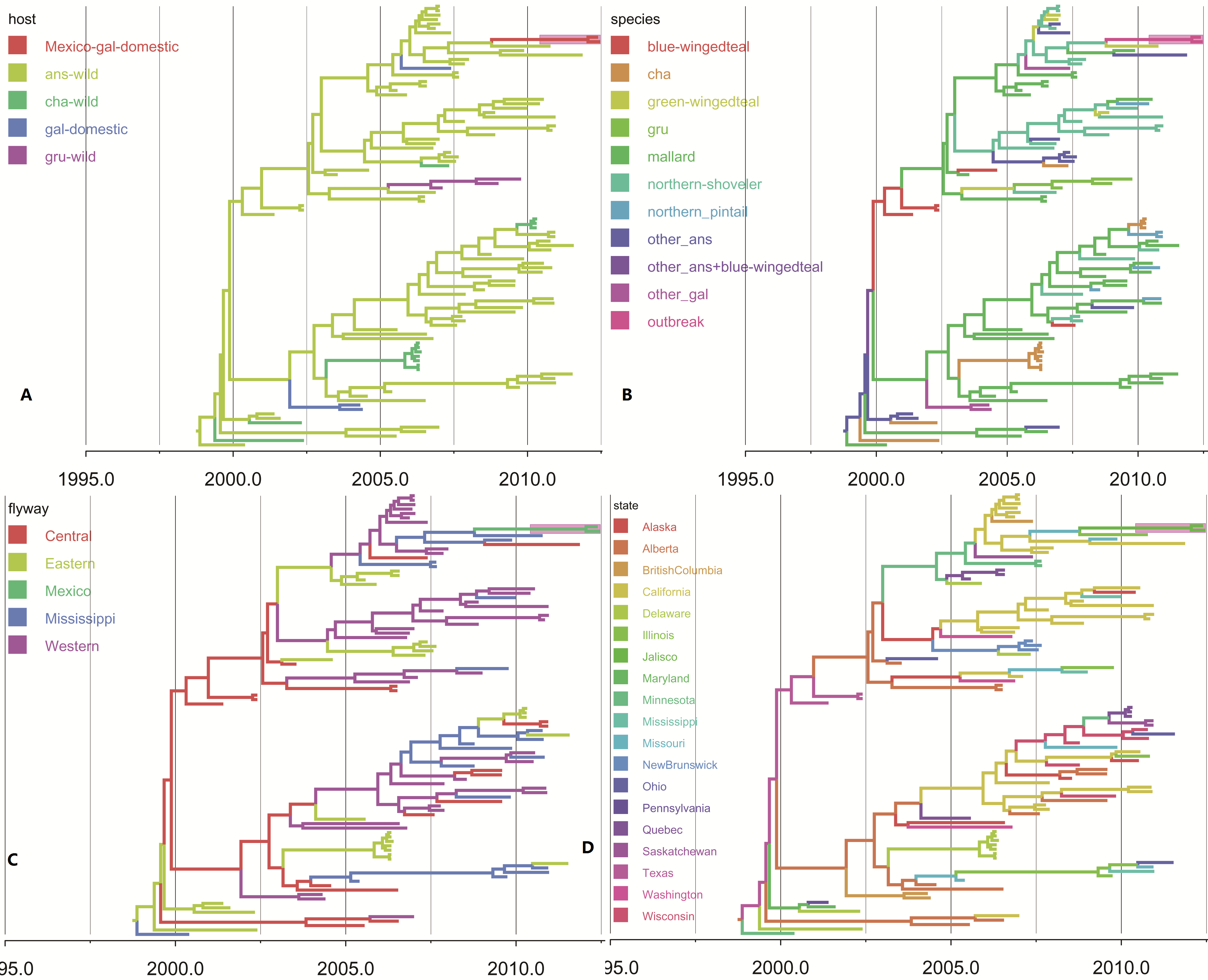

Supplement: Figure S2 — Maximum clade credibility (MCC) phylogenies for the NA segment. Temporally structured maximum clade credibility (mcc) time-scaled phylogenetic tree showing the evolution of NA gene of avian influenza A virus isolated from North American wild birds for each individual gene dataset. Ancestral state (A: host order; B: host species; location; C: flyway; D: state) changes recovered from the discrete trait analyses are indicated by color changes at tree nodes. Mexican outbreak strains are highlighted with pink. A: Host order. Five host orders are labelled on HA tree: wild birds of the order Anseriformes (ans-wild); wild birds of the order Charadriiformes (cha-wild); wild birds of the order Passeriformes (pas-wild); domestic birds of the order Galliformes and Mexico H7N3 outbreak in the order Galliformes (gal-domestic-Mexico). B: Host species. Wild Anseriformes are classified into the five main species and a group comprising the other rarer species of Anseriformes in this study: mallard (Anas platyrhynchos), northern pintail (Anasacuta), northern shoveller, blue-winged teal, green-winged teal and other Anseriformes (other ans); The order Galliformes are shown as “outbreak” (the H7N3 Mexico outbreak) and “other_gal”; The other orders are shown as: Charadriiformes (cha) and Galliformes (gal), Gruiformes (gru) and Passeriformes (pas). C: Flyway. Four specific North American flyways are labelled on HA tree: the Atlantic (Eastern), Mississippi, Central, and Pacific (Western). D: State. 22 states and provinces of the viral sample locations are labelled on HA tree. The same color code also applies to Figures S3, S4, S5, S6, S7, S8. (TIF) [file pone.0107330.s002.tif]

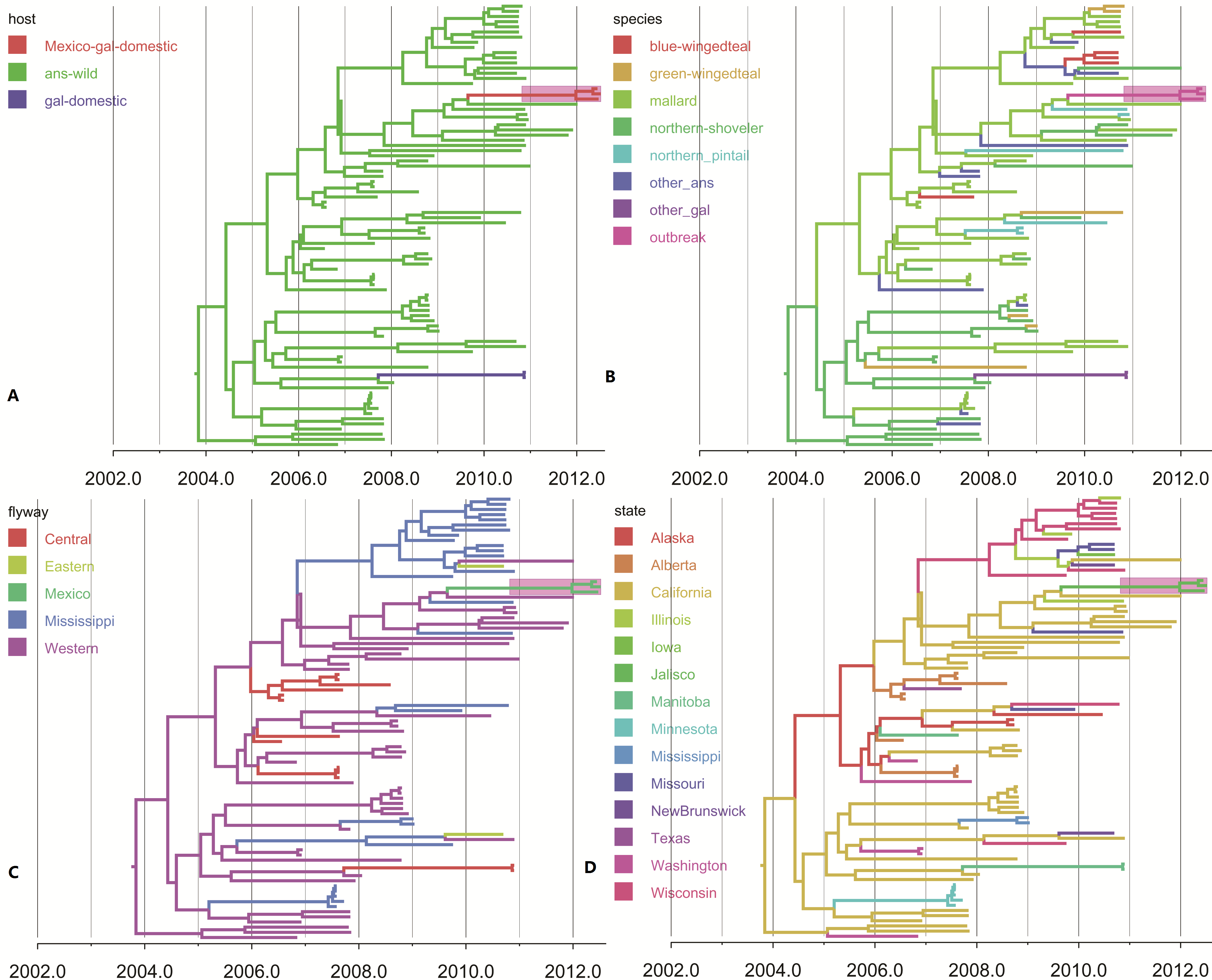

Supplement: Figure S3 — MCC phylogenies for the PB2 segment. (TIF) [file pone.0107330.s003.tif]

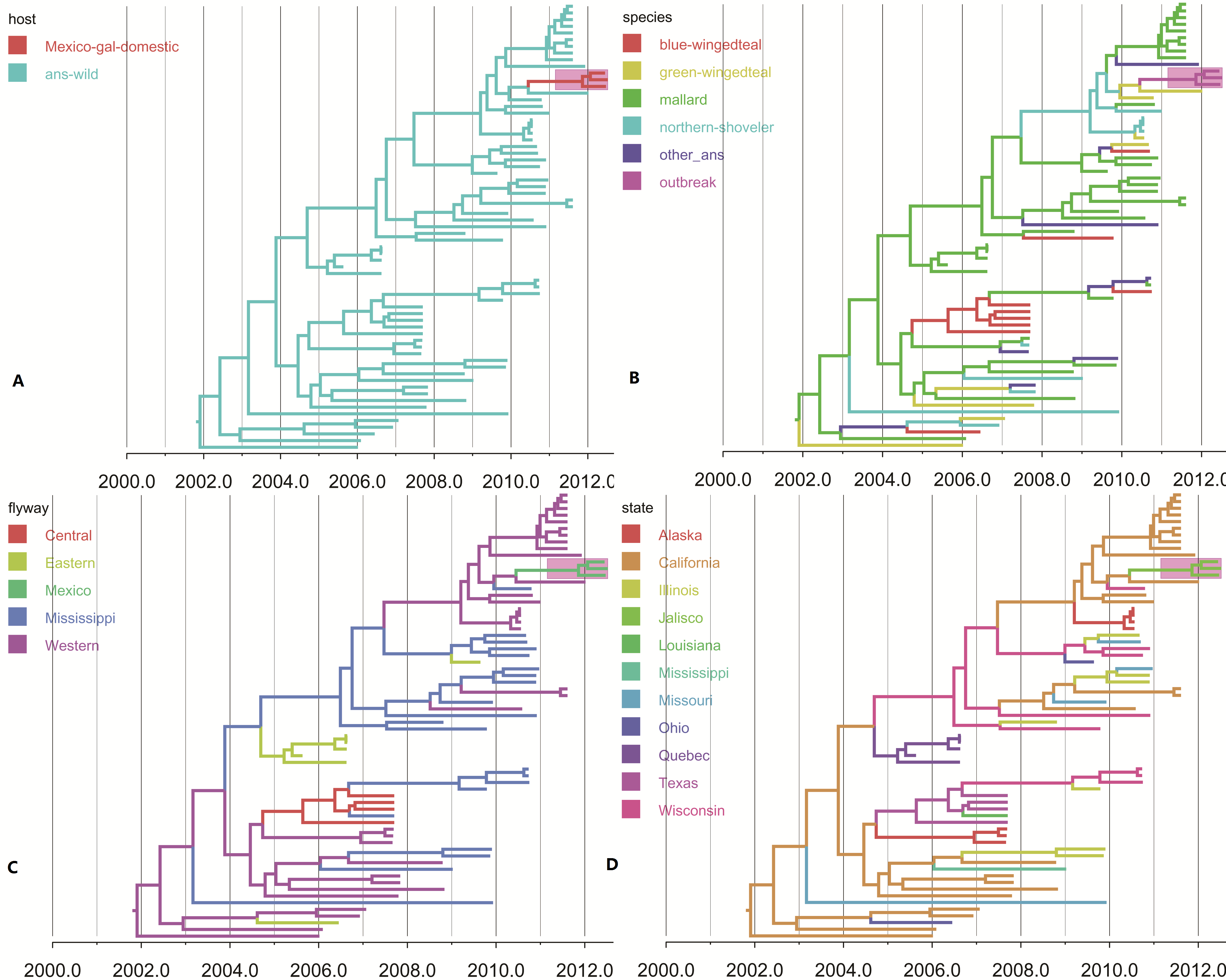

Supplement: Figure S4 — MCC phylogenies for the PB1 segment. (TIF) [file pone.0107330.s004.tif]

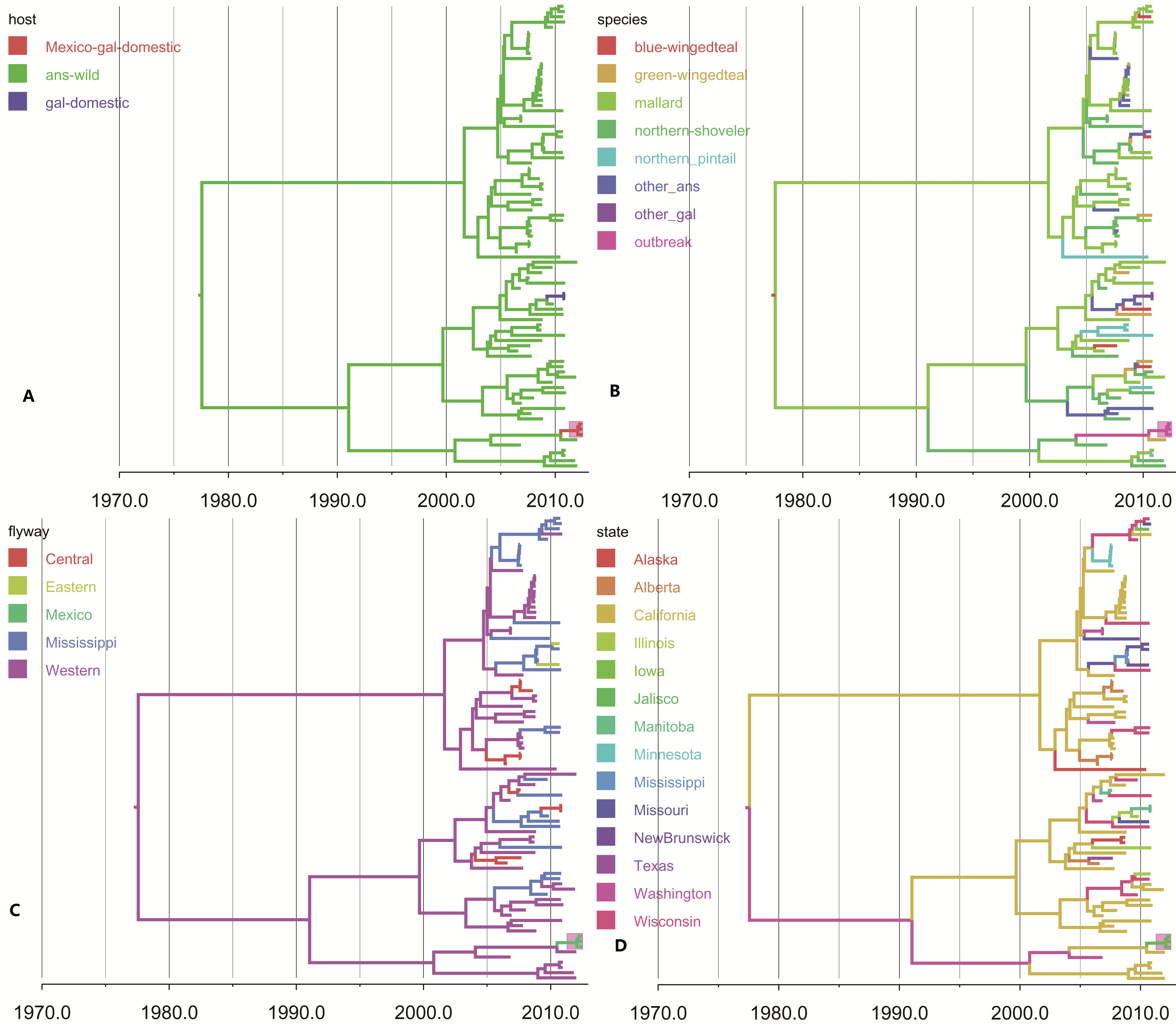

Supplement: Figure S5 — MCC phylogenies for the PA segment. (TIF) [file pone.0107330.s005.tif]

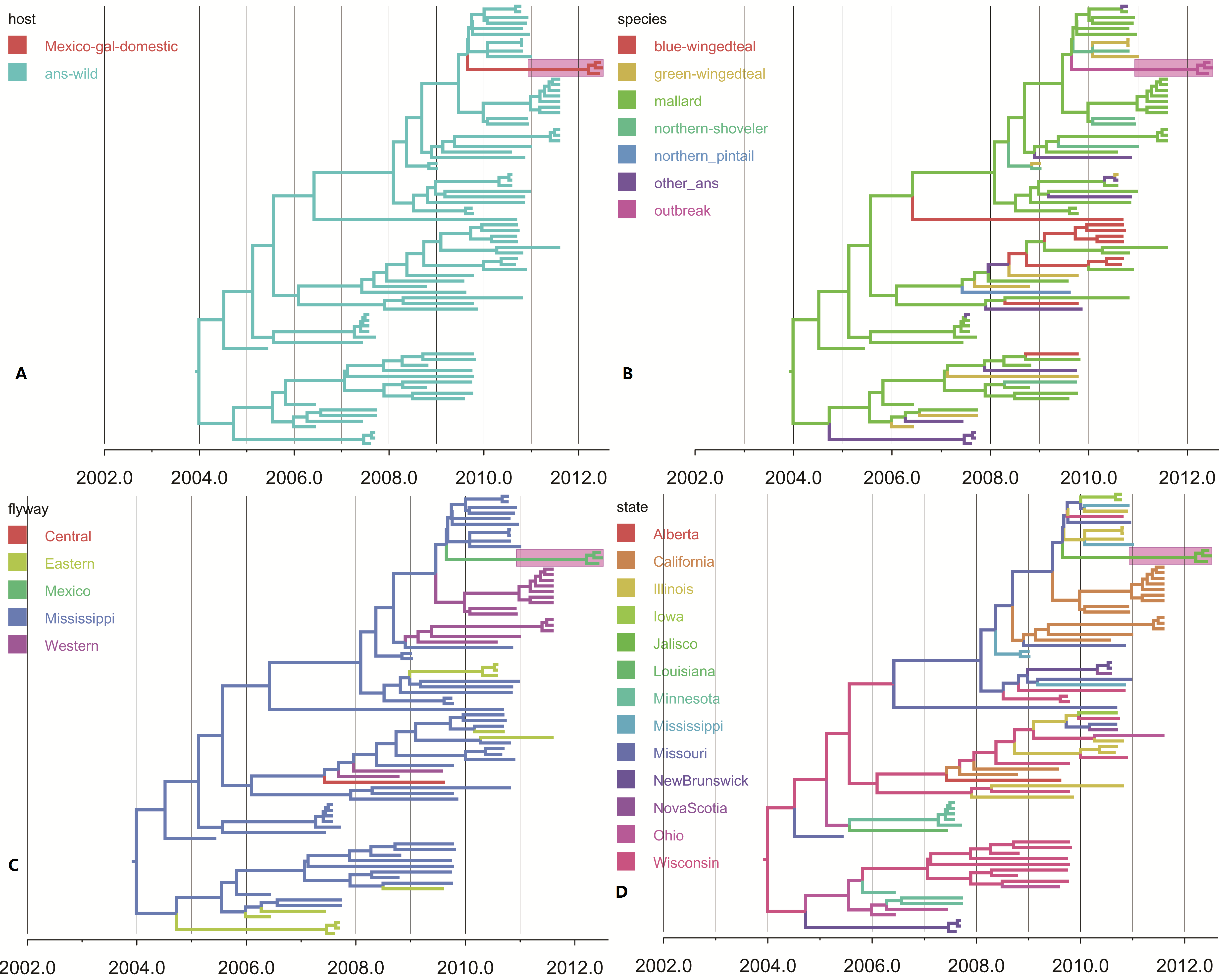

Supplement: Figure S6 — MCC phylogenies for the NP segment. (TIF) [file pone.0107330.s006.tif]

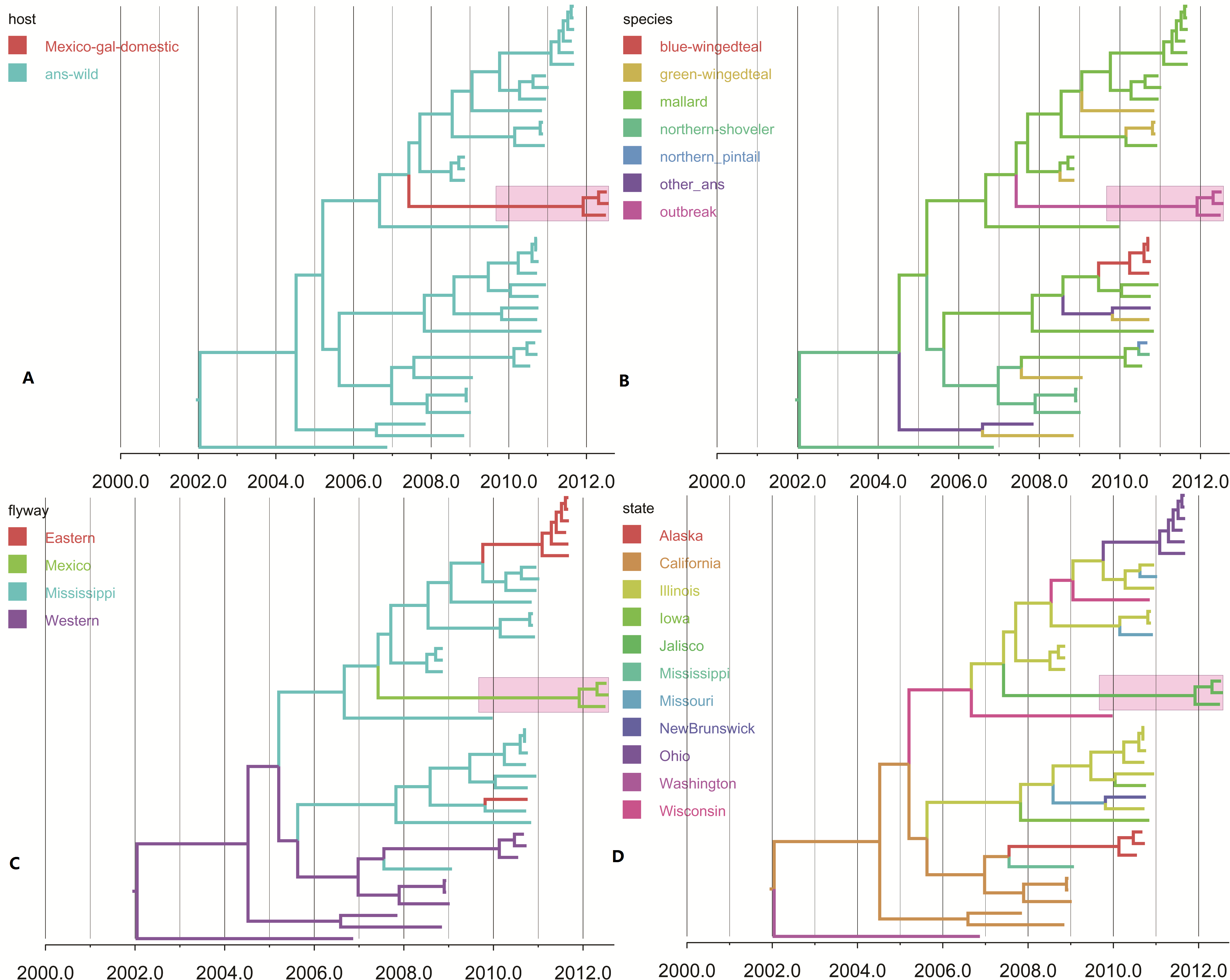

Supplement: Figure S7 — MCC phylogenies for the M segment. (TIF) [file pone.0107330.s007.tif]

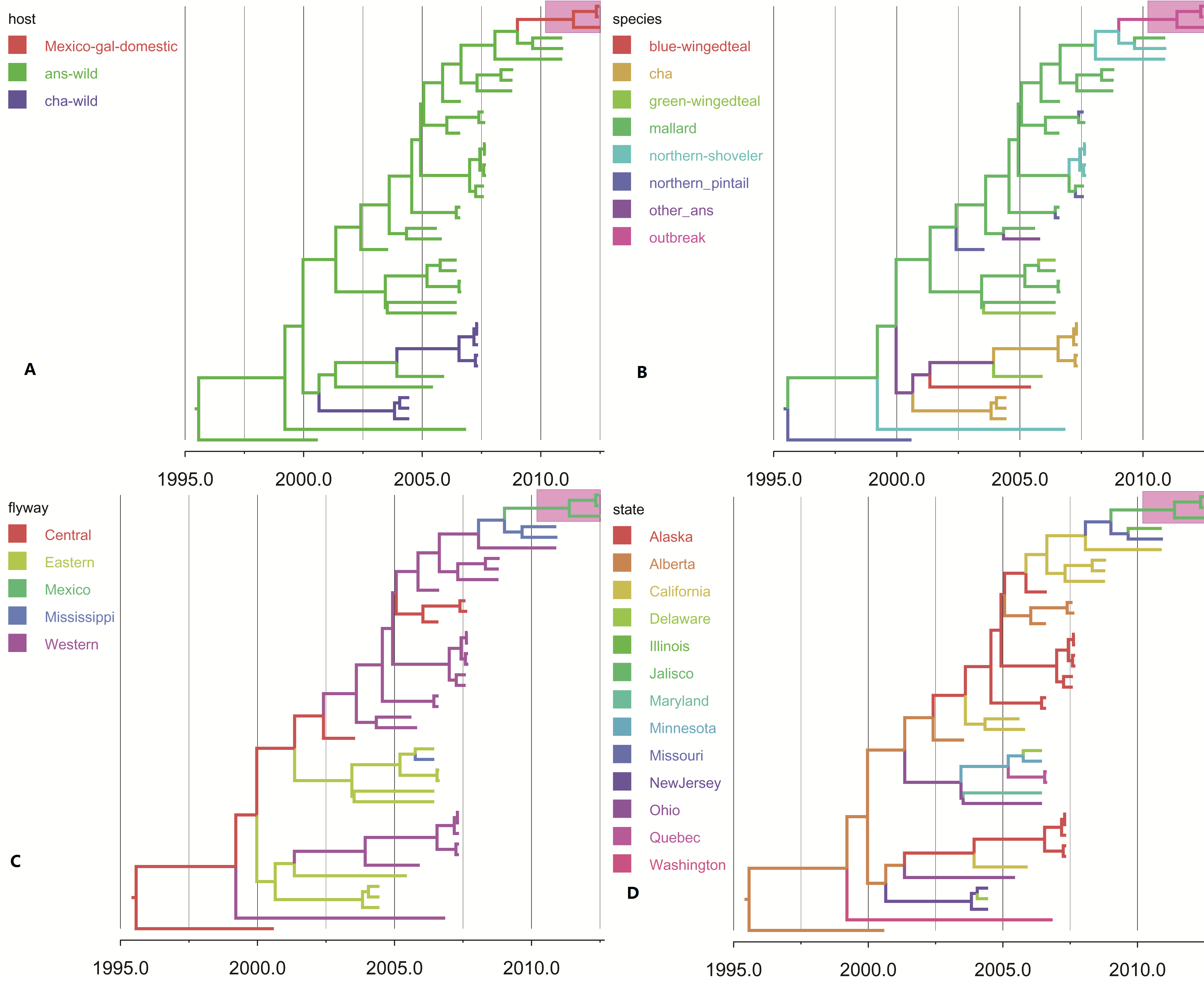

Supplement: Figure S8 — MCC phylogenies for the NS segment. (TIF) [file pone.0107330.s008.tif]

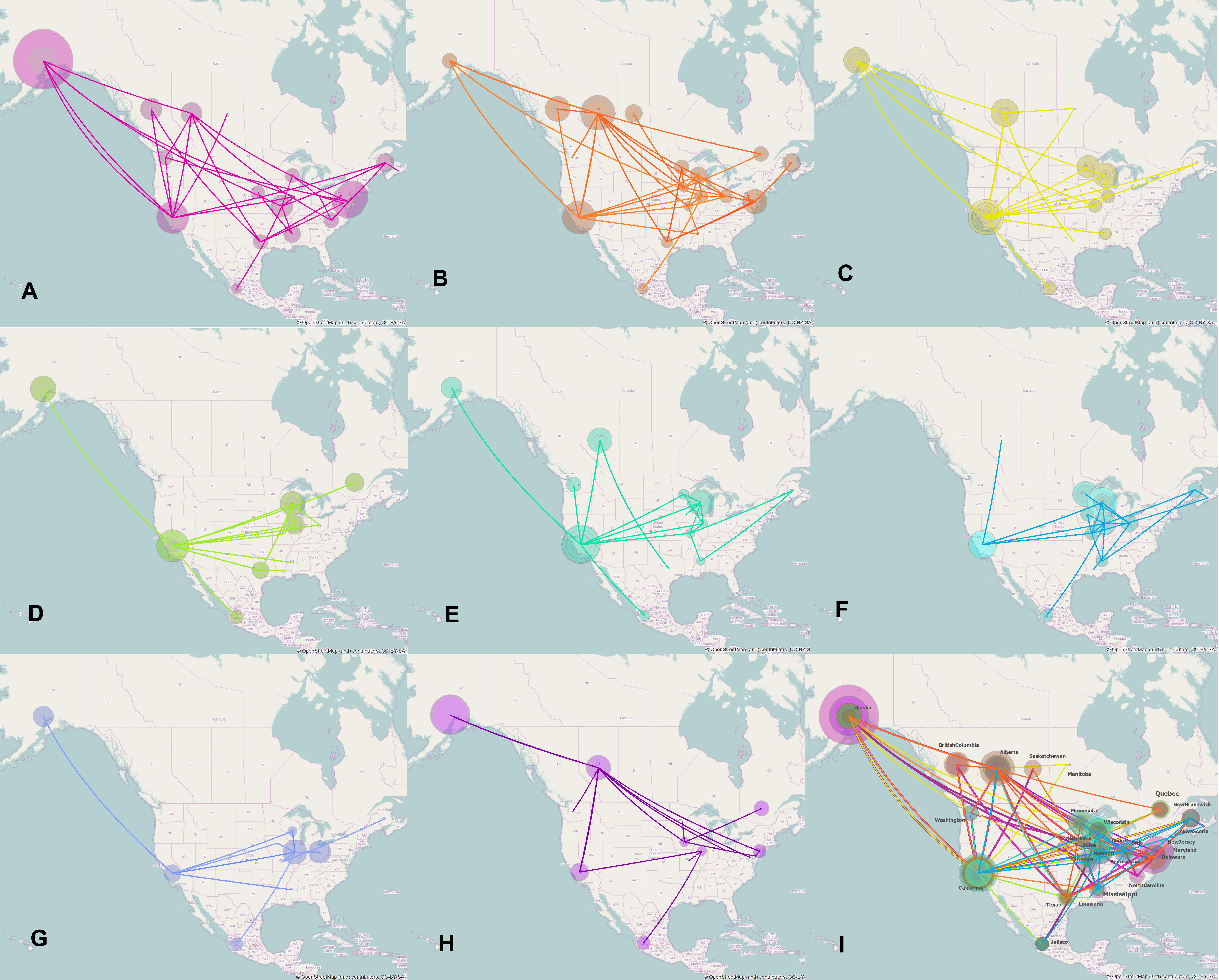

Supplement: Figure S9 — Spatial diffusion of AIV segments of the Mexico outbreak. Panels represent eight segments separately (A: HA, B: NA, C: PB2, D: PB1, E: PA, F: NP, G: M, H: NS) and I represents the spatial transmission of all 8 segments jointly. The Map source for this figure was OpenStreetMap (http://www.openstreetmap.org/). Plotted lines are the branches of the MCC trees of different segments, distinguished by color; the size of each circle represents the number of lineages with that location state. (TIF) [file pone.0107330.s009.tif]

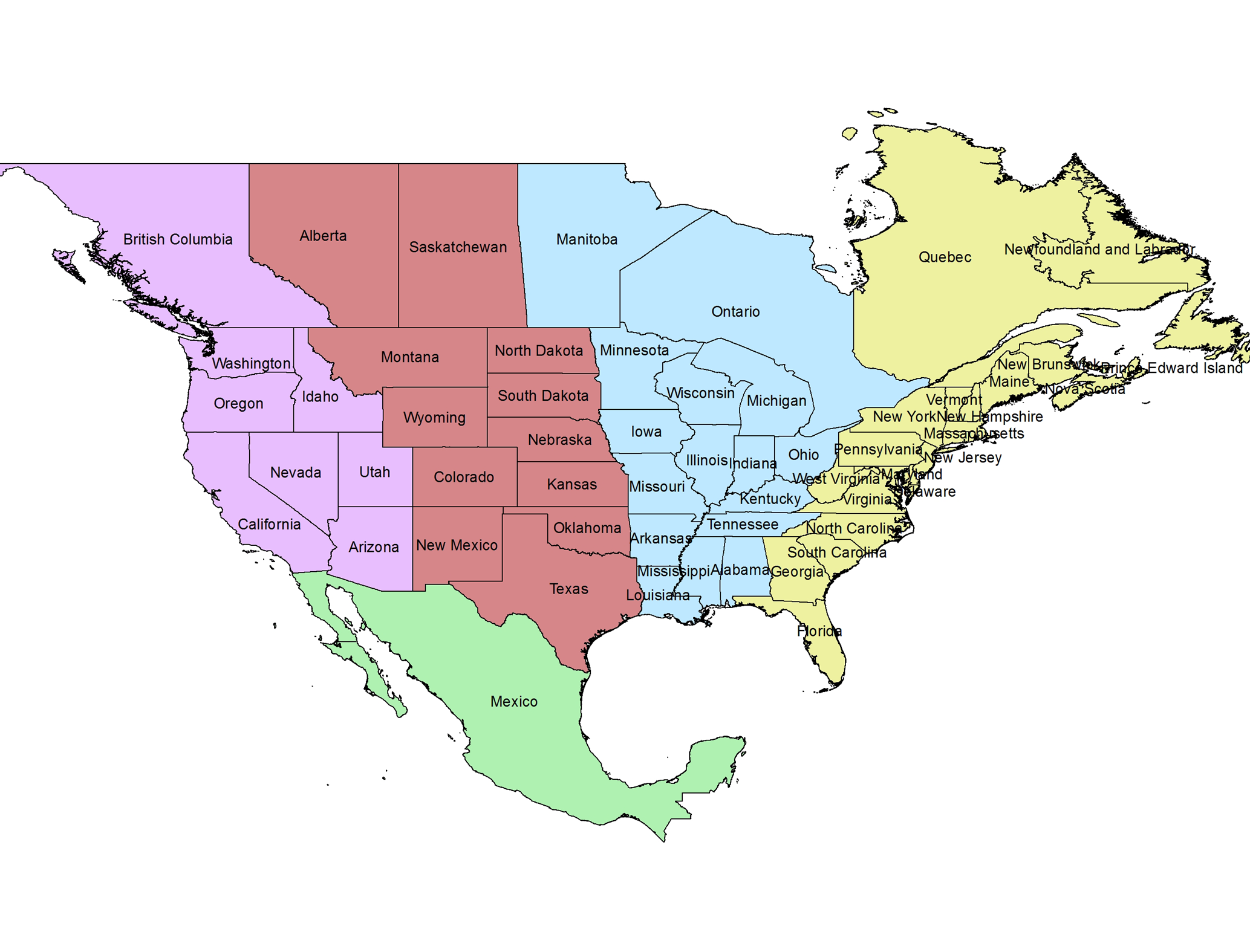

Supplement: Figure S10 — North America states and provinces categorized according to flyways. Provinces and states which belong to Pacific flyways are colored in purple; Central flyways are colored in red; Mississippi flyways are colored in blue; Atlantic flyways are colored in yellow; and Mexico is colored in green. Those colors on the map are the same as the colored branches in the flyway discrete phylogenetic tree in Figure 2 C. (TIF) [file pone.0107330.s010.tif]

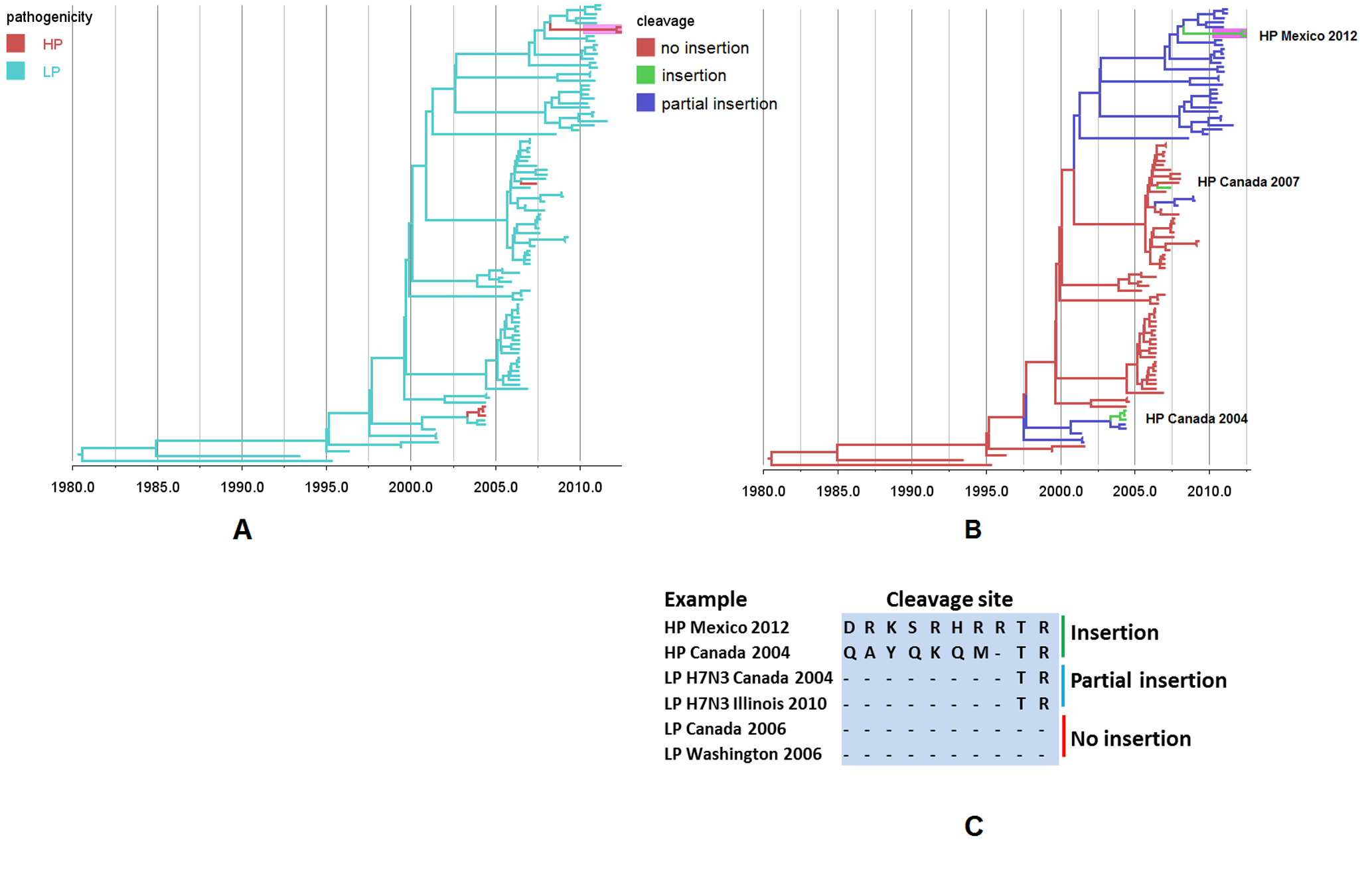

Supplement: Figure S11 — Virulence evolving on the MCC phylogeny of the HA segment. HA cleavage site insertions are classified as follows: 1) Insertion: which are multibasic insertions (insertion) which only being found in HP AIV, including HP H7N3 outbreaks strains in Mexico 2012 and in Canada 2004 and 2007; 2) Partial insertion: LP AIV which has a partial insertion of 2 amino acids; 3) No insertion: other LP AIV with no insertion in the cleavage site. (A): Pathogenicity changes (according to high pathogenicity (HP) and low pathogenicity (LP)) are indicated by color changes in the tree; Cleavage site changes (with insertion, partial insertions and no insertions (C) are indicated by color changes at tree nodes. (TIF) [file pone.0107330.s011.tif]
