# Supplementary material for: Determining the Phylogenetic and Phylogeographic Origin of Highly Pathogenic Avian Influenza (H7N3) in Mexico
Source: PLoS One. 2014 Sep 16;9(9):e107330. doi: 10.1371/journal.pone.0107330 (PMC4165766; doi:10.1371/journal.pone.0107330)
Supplement: Table S1 — Host orders distribution of 427 AIV sequences. (DOCX) [file pone.0107330.s012.docx]

Table S1. Host order states and distribution of 427 AIV sequences

| **Number** | **Host** | **Numbers** |
| --- | --- | --- |
| 1 | ans-wild | 366 |
| 2 | cha-wild | 45 |
| 3 | pas-wild | 2 |
| 4 | gru-wild | 3 |
| 5 | gal-domestic | 8 |
| 6 | Mexico-gal-domestic | 3 |
