# Supplementary material for: Determining the Phylogenetic and Phylogeographic Origin of Highly Pathogenic Avian Influenza (H7N3) in Mexico
Source: PLoS One. 2014 Sep 16;9(9):e107330. doi: 10.1371/journal.pone.0107330 (PMC4165766; doi:10.1371/journal.pone.0107330)
Supplement: Table S2 — Host species (Anseriformes only) distribution of 366 AIV sequences. (DOCX) [file pone.0107330.s013.docx]

Table S2. Host species (Anseriformes only) states and the distribution of 366 AIV sequences

| **Number** | **Host species (ans)** | **Numbers** |
| --- | --- | --- |
| 1 | mallard | 163 |
| 2 | Northern shoveler | 67 |
| 3 | Northern pintail | 24 |
| 4 | Green winged teal | 39 |
| 5 | Blue winged teal | 29 |
| 6 | American black duck | 9 |
| 7 | Others ans | 35 |
