# Supplementary material for: Determining the Phylogenetic and Phylogeographic Origin of Highly Pathogenic Avian Influenza (H7N3) in Mexico
Source: PLoS One. 2014 Sep 16;9(9):e107330. doi: 10.1371/journal.pone.0107330 (PMC4165766; doi:10.1371/journal.pone.0107330)
Supplement: Table S3 — Location (state/province) distribution of 427 AIV sequences. (DOCX) [file pone.0107330.s014.docx]

Table S3.Location (State/province) and the distribution of 427 AIV sequences

| **Number** | **State** | **Flyway** | **Numbers** |
| --- | --- | --- | --- |
| 1 | Alaska | Pacific | 44 |
| 2 | Alberta | Central | 28 |
| 3 | BritishColumbia | Pacific | 5 |
| 4 | California | Pacific | 114 |
| 5 | Delaware | Atlantic | 26 |
| 6 | Illinois | Mississippi | 28 |
| 7 | Iowa | Mississippi | 5 |
| 8 | Jalisco | Outbreak | 3 |
| 9 | Louisiana | Mississippi | 2 |
| 10 | Manitoba | Mississippi | 3 |
| 11 | Maryland | Atlantic | 4 |
| 12 | Minnesota | Mississippi | 11 |
| 13 | Mississippi | Mississippi | 14 |
| 14 | Missouri | Mississippi | 19 |
| 15 | Nebraska | Central | 2 |
| 16 | NewBrunswick | Atlantic | 10 |
| 17 | NewJersey | Atlantic | 15 |
| 18 | NorthCarolina | Atlantic | 3 |
| 19 | NovaScotia | Atlantic | 3 |
| 20 | Ohio | Mississippi | 18 |
| 21 | Pennsylvania | Atlantic | 1 |
| 22 | Quebec | Atlantic | 12 |
| 23 | Saskatchewan | Central | 3 |
| 24 | Texas | Central | 10 |
| 25 | Washington | Pacific | 9 |
| 26 | Wisconsin | Mississippi | 35 |
