# Supplementary material for: Determining the Phylogenetic and Phylogeographic Origin of Highly Pathogenic Avian Influenza (H7N3) in Mexico
Source: PLoS One. 2014 Sep 16;9(9):e107330. doi: 10.1371/journal.pone.0107330 (PMC4165766; doi:10.1371/journal.pone.0107330)
Supplement: Table S4 — Flyway distribution of 427 AIV sequences. (DOCX) [file pone.0107330.s015.docx]

Table S4. Flyway and the distribution of 427 AIV sequences

| **Number** | **Flyway** | **Numbers** |
| --- | --- | --- |
| 1 | Atlantic | 76 |
| 2 | Central | 44 |
| 3 | Mississippi | 132 |
| 4 | Pacific | 172 |
| 5 | Outbreak | 3 |
