# Supplementary material for: Determining the Phylogenetic and Phylogeographic Origin of Highly Pathogenic Avian Influenza (H7N3) in Mexico
Source: PLoS One. 2014 Sep 16;9(9):e107330. doi: 10.1371/journal.pone.0107330 (PMC4165766; doi:10.1371/journal.pone.0107330)
Supplement: Table S5 — Transmission rates of host orders and Bayes Factor support. (DOCX) [file pone.0107330.s016.docx]

Table S5. Transmission rates of host orders and the Bayes Factor support

| **Transition** | | **Mean rate** | **Indicator** | **BF** |
| --- | --- | --- | --- | --- |
| Charadriiformes (wild) | Passeriformes (wild) | 0.07 | 1 | >100 |
| Anseriformes | Charaiformes (wild) | 0.02 | 1 | >100 |
| Anseriformes (wild) | Galliformes outbreak | 0.02 | 0.99 | >100 |
| Anseriformes (wild) | Galliformes (domestic) | 0.01 | 0.99 | >100 |

States=6 (host)

Indicator cutoff (for BF = 3.0) = 0.65
