# Supplementary material for: Determining the Phylogenetic and Phylogeographic Origin of Highly Pathogenic Avian Influenza (H7N3) in Mexico
Source: PLoS One. 2014 Sep 16;9(9):e107330. doi: 10.1371/journal.pone.0107330 (PMC4165766; doi:10.1371/journal.pone.0107330)
Supplement: Table S6 — Transmission rates of host species and Bayes Factor support. (DOCX) [file pone.0107330.s017.docx]

Table S6. Transmission rates of host species and the Bayes Factor support

| **Transition** | | **Mean rate** | **Indicator** | **BF** |
| --- | --- | --- | --- | --- |
| other_ans | blue-wingedteal | 0.68 | 0.85 | 19 |
| northern-shoveler | green-wingedteal | 2.13 | 1 | >100 |
| other_ans | green-wingedteal | 1.57 | 0.99 | >100 |
| outbreak | green-wingedteal | 0.15 | 0.67 | 6 |
| northern-shoveler | mallard | 0.38 | 0.99 | >100 |
| northern_pintail | mallard | 0.16 | 0.99 | >100 |
| other_ans | mallard | 0.41 | 1 | >100 |

State=9 (host species)

Indicator cutoff (for BF = 3.0) = 0.49
