# Supplementary material for: Determining the Phylogenetic and Phylogeographic Origin of Highly Pathogenic Avian Influenza (H7N3) in Mexico
Source: PLoS One. 2014 Sep 16;9(9):e107330. doi: 10.1371/journal.pone.0107330 (PMC4165766; doi:10.1371/journal.pone.0107330)
Supplement: Table S7 — Transmission rates of flyway and Bayes Factor support. (DOCX) [file pone.0107330.s018.docx]

Table S7. Transmission rates of flyways and the Bayes Factor support

| **Transition** | | **Mean rate** | **Indicator** | **BF** |
| --- | --- | --- | --- | --- |
| Atlantic | Central | 0.25 | 1 | >100 |
| Pacific | Central | 0.18 | 1 | >100 |
| Mississippi | Atlantic | 0.17 | 1 | >100 |
| Mexico | Mississippi | 0.05 | 0.98 | 86 |
| Pacific | Mississippi | 0.22 | 1 | >100 |
| Mexico | Pacific | 0.02 | 0.73 | 4 |
| Mexico | Central | 0.04 | 0.73 | 4 |
| Central | Mississippi | 0.05 | 0.73 | 4 |
| Pacific | Atlantic | 0.04 | 0.72 | 3 |

States=5 (flyway)

Indicator cutoff (for BF = 3.0) = 0.72
