# Supplementary material for: Determining the Phylogenetic and Phylogeographic Origin of Highly Pathogenic Avian Influenza (H7N3) in Mexico
Source: PLoS One. 2014 Sep 16;9(9):e107330. doi: 10.1371/journal.pone.0107330 (PMC4165766; doi:10.1371/journal.pone.0107330)
Supplement: Table S8 — Transmission rates of location (state/province) and the Bayes Factor support. (DOCX) [file pone.0107330.s019.docx]

Table S8. Transmission rates of location (state/province) and the Bayes Factor support

| **Transition** | | **Mean rate** | **Indicator** | **BF** |
| --- | --- | --- | --- | --- |
| Jalisco | Alaska | 0.59 | 0.99 | >100 |
| Jalisco | Alberta | 1.07 | 1 | >100 |
| Jalisco | California | 0.59 | 1 | >100 |
| Jalisco | Illinois | 1.61 | 1 | >100 |
| Jalisco | Minnesota | 0.33 | 0.7 | 21 |
| Jalisco | Missouri | 2.26 | 1 | >100 |
| Jalisco | New Brunswick | 0.47 | 0.82 | 26 |
| Jalisco | Ohio | 0.66 | 1 | >100 |
| Jalisco | Quebec | 0.31 | 0.84 | 30 |
| Jalisco | Washington | 0.54 | 0.78 | 21 |
| Jalisco | Wisconsin | 1.39 | 1 | >100 |

States=26 (locations)

Indicator cutoff (for BF = 3.0) = 0.34
