# Supplementary material for: Determining the Phylogenetic and Phylogeographic Origin of Highly Pathogenic Avian Influenza (H7N3) in Mexico
Source: PLoS One. 2014 Sep 16;9(9):e107330. doi: 10.1371/journal.pone.0107330 (PMC4165766; doi:10.1371/journal.pone.0107330)
Supplement: Table S9 — AIV sequences being used in this study. The strain name and subtype, host, location, flyway and the segments being selected in the AIV dataset. Details of the 427 North American AIV sequences used in this analysis. Strain name, subtype, host orders, isolated state/provinces and the correspondent flyways are listed. (DOCX) [file pone.0107330.s020.docx]

Table S9 AIV sequences being used in this study.The strain name and subtype, host, location , flyway and the segments being selected in the AIV dataset

| **Name** | **Subtype** | **Host** | **State** | **Flyway** | **Segment** | | | | | | | |
| --- | --- | --- | --- | --- | --- | --- | --- | --- | --- | --- | --- | --- |
| A/mallard/Missouri/10MO0253/2010 | H1N2 | ans-wild | Missouri | Mississippi | PB2 |  | PA |  |  |  |  |  |
| A/northernshoveler/California/2810/2011 | H11N2 | ans-wild | California | Pacific | PB2 |  | PA |  |  |  |  |  |
| A/mallard/California/2961/2011 | H2N3 | ans-wild | California | Pacific | PB2 |  | PA |  |  |  |  |  |
| A/northernshoveler/California/3183/2010 | H11N2 | ans-wild | California | Pacific | PB2 |  | PA |  |  |  |  | NS |
| A/Mexico/InDRE7218/2012 | H7N3 | Mexico-gal-domestic | Jalisco | Mexico | PB2 | PB1 | PA |  | NP | NA | M | NS |
| A/chicken/Jalisco/CPA1/2012 | H7N3 | Mexico-gal-domestic | Jalisco | Mexico | PB2 | PB1 | PA | HA | NP | NA | M | NS |
| A/chicken/Jalisco/12283/2012 | H7N3 | Mexico-gal-domestic | Jalisco | Mexico | PB2 | PB1 | PA | HA | NP |  | M | NS |
| A/northernpintail/Illinois/10OS3959/2010 | H7N3 | ans-wild | Illinois | Mississippi | PB2 |  | PA | HA |  | NA |  |  |
| A/northernpintail/California/3466/2010 | H1N3 | ans-wild | California | Pacific | PB2 |  | PA |  |  | NA |  |  |
| A/mallard/California/3569/2010 | H1N3 | ans-wild | California | Pacific | PB2 |  | PA |  |  | NA |  |  |
| A/mallard/California/198/2012 | H11N9 | ans-wild | California | Pacific | PB2 |  | PA |  |  |  |  |  |
| A/green-wingedteal/California/8326/2008 | H1N2 | ans-wild | California | Pacific | PB2 |  | PA |  | NP |  | M |  |
| A/northernshoveler/California/HKWF96/2007 | H10N7 | ans-wild | California | Pacific | PB2 |  | PA |  |  |  |  |  |
| A/northernshoveler/California/9680/2008 | H6N2 | ans-wild | California | Pacific | PB2 |  | PA |  |  |  |  |  |
| A/green-wingedteal/California/8612/2008 | H6N1 | ans-wild | California | Pacific | PB2 |  | PA |  |  |  |  |  |
| A/northernshoveler/California/8673/2008 | H6N1 | ans-wild | California | Pacific | PB2 |  | PA |  |  |  |  |  |
| A/greaterwhite-frontedgoose/California/6365/2008 | H6N1 | ans-wild | California | Pacific | PB2 |  | PA |  |  |  |  |  |
| A/mallard/California/8322/2008 | H6N1 | ans-wild | California | Pacific | PB2 |  | PA |  |  |  |  |  |
| A/mallard/California/8462/2008 | H6N1 | ans-wild | California | Pacific | PB2 |  |  |  |  |  |  |  |
| A/northernshoveler/California/44363-082/2007 | H11N9 | ans-wild | California | Pacific | PB2 |  | PA |  |  |  |  |  |
| A/Americangreen-wingedteal/Mississippi/09OS046/2009 | H7N7 | ans-wild | Mississippi | Mississippi | PB2 |  | PA | HA | NP |  | M |  |
| A/northernshoveler/Mississippi/09OS643/2009 | H7N7 | ans-wild | Mississippi | Mississippi | PB2 |  | PA | HA | NP |  |  |  |
| A/Americanwigeon/California/3180/2010 | H5N2 | ans-wild | California | Pacific | PB2 |  | PA |  |  |  |  |  |
| A/northernshoveler/California/JN587/2006 | H10N3 | ans-wild | California | Pacific | PB2 | PB1 | PA |  |  | NA |  |  |
| A/Americanwigeon/California/HKWF450/2007 | H4N7 | ans-wild | California | Pacific | PB2 |  | PA |  |  |  |  |  |
| A/northernshoveler/California/HKWF392sm/2007 | H10N7 | ans-wild | California | Pacific | PB2 |  | PA |  |  |  |  |  |
| A/mallard/Minnesota/Sg-00056/2007 | H10N7 | ans-wild | Minnesota | Mississippi | PB2 |  | PA |  | NP |  |  |  |
| A/mallard/Minnesota/Sg-00057/2007 | H10N7 | ans-wild | Minnesota | Mississippi | PB2 |  | PA |  | NP |  |  |  |
| A/ring-neckedduck/Minnesota/Sg-00068/2007 | H10N7 | ans-wild | Minnesota | Mississippi | PB2 |  | PA |  | NP |  |  |  |
| A/mallard/Minnesota/Sg-00065/2007 | H10N7 | ans-wild | Minnesota | Mississippi | PB2 |  | PA |  | NP |  |  |  |
| A/mallard/Minnesota/Sg-00195/2007 | H10N3 | ans-wild | Minnesota | Mississippi | PB2 |  | PA |  | NP | NA |  |  |
| A/turkey/MB/FAV11/2010 | H5N2 | gal-domestic | Manitoba | Mississippi | PB2 |  | PA |  |  |  |  |  |
| A/turkey/MB/FAV10/2010 | H5N2 | gal-domestic | Manitoba | Mississippi | PB2 |  | PA |  |  |  |  |  |
| A/northernshoveler/California/HKWF2031/2008 | H7N3 | ans-wild | California | Pacific | PB2 |  | PA | HA |  | NA |  |  |
| A/northernpintail/California/2548/2010 | H11N2 | ans-wild | California | Pacific | PB2 |  | PA |  |  |  |  |  |
| A/northernshoveler/California/138/2012 | H1N1 | ans-wild | California | Pacific | PB2 |  | PA |  |  |  |  |  |
| A/northernpintail/InteriorAlaska/8BM3669/2008 | H4N6 | ans-wild | Alaska | Pacific | PB2 |  | PA |  |  |  |  |  |
| A/northernpintail/InteriorAlaska/8BM3736/2008 | H12N5 | ans-wild | Alaska | Pacific | PB2 |  | PA |  |  |  |  |  |
| A/northernshoveler/California/HKWF608/2007 | H10N7 | ans-wild | California | Pacific | PB2 |  | PA |  |  |  |  |  |
| A/northernshoveler/California/3628/2011 | H10N3 | ans-wild | California | Pacific | PB2 | PB1 | PA |  |  | NA |  |  |
| A/northernshoveler/Washington/44249-664/2006 | H7N3 | ans-wild | Washington | Pacific | PB2 |  | PA | HA |  | NA |  |  |
| A/mallard/California/6524/2008 | H12N5 | ans-wild | California | Pacific | PB2 |  | PA |  |  |  |  |  |
| A/mallard/Wisconsin/2549/2009 | H3N2 | ans-wild | Wisconsin | Mississippi | PB2 |  | PA |  |  |  |  |  |
| A/mallard/California/3188/2010 | H6N8 | ans-wild | California | Pacific | PB2 |  | PA |  |  |  |  |  |
| A/mallard/NewBrunswick/00854/201 | H4N6 | ans-wild | NewBrunswick | Atlantic | PB2 |  | PA |  |  |  |  |  |
| A/mallard/Manitoba/23912/2007 | H4N7 | ans-wild | Manitoba | Mississippi | PB2 |  | PA |  |  |  |  |  |
| A/mallard/Wisconsin/2575/2009 | H3N2 | ans-wild | Wisconsin | Mississippi | PB2 |  | PA |  | NP |  |  |  |
| A/mallard/Wisconsin/10OS2889/2010 | H11N9 | ans-wild | Wisconsin | Mississippi | PB2 |  | PA |  |  |  |  |  |
| A/Americangreen-wingedteal/Wisconsin/10OS2955/2010 | H5N2 | ans-wild | Wisconsin | Mississippi | PB2 |  | PA |  |  |  |  |  |
| A/Americangreen-wingedteal/Illinois/10OS3662/2010 | H11N2 | ans-wild | Illinois | Mississippi | PB2 |  | PA |  |  |  |  |  |
| A/mallard/Wisconsin/10OS2538/2010 | H3N2 | ans-wild | Wisconsin | Mississippi | PB2 |  | PA |  |  |  |  |  |
| A/mallard/Wisconsin/10OS2909/2010 | H3N8 | ans-wild | Wisconsin | Mississippi | PB2 |  | PA |  |  |  |  |  |
| A/mallard/Wisconsin/2653/2009 | H4N6 | ans-wild | Wisconsin | Mississippi | PB2 |  | PA |  |  |  |  |  |
| A/mallard/Wisconsin/10OS3169/2010 | H4N9 | ans-wild | Wisconsin | Mississippi | PB2 |  | PA |  |  |  |  |  |
| A/Americanblackduck/Illinois/3854/2009 | H11N9 | ans-wild | Illinois | Mississippi | PB2 |  | PA |  | NP |  |  |  |
| A/blue-wingedteal/Wisconsin/10OS3092/2010 | H3N6 | ans-wild | Wisconsin | Mississippi | PB2 |  | PA |  | NP |  |  |  |
| A/mallard/Wisconsin/10OS4194/2010 | H11N9 | ans-wild | Wisconsin | Mississippi | PB2 | PB1 | PA |  | NP |  |  |  |
| A/Americanblackduck/NewBrunswick/00998/2010 | H12N6 | ans-wild | NewBrunswick | Atlantic | PB2 |  | PA |  |  |  | M |  |
| A/blue-wingedteal/Missouri/10MO003/2010 | H4N6 | ans-wild | Missouri | Mississippi | PB2 |  | PA |  | NP |  |  |  |
| A/blue-wingedteal/Missouri/10MO0011/2010 | H3N6 | ans-wild | Missouri | Mississippi | PB2 | PB1 | PA |  |  |  |  |  |
| A/blue-wingedteal/Iowa/10OS2639/2010 | H3N8 | ans-wild | Iowa | Mississippi | PB2 |  | PA |  | NP |  |  |  |
| A/northernshoveler/Washington/44249-783/2006 | H7N3 | ans-wild | Washington | Pacific | PB2 |  | PA | HA |  |  |  |  |
| A/northernshoveler/Washington/44249-752/2006 | H7N3 | ans-wild | Washington | Pacific | PB2 |  | PA |  |  |  |  |  |
| A/northernshoveler/California/HKWF1325/2007 | H8N4 | ans-wild | California | Pacific | PB2 |  | PA |  |  |  |  |  |
| A/westerngrebe/Washington/20569-004/2007 | H1N2 | ans-wild | Washington | Pacific | PB2 |  | PA |  |  |  |  |  |
| A/Americanwigeon/California/HKWF295/2007 | H6N5 | ans-wild | California | Pacific | PB2 |  | PA |  |  |  |  |  |
| A/mallard/California/11100/2008 | H11N2 | ans-wild | California | Pacific | PB2 |  | PA |  |  |  |  |  |
| A/mallard/Alberta/107/2007 | H3N8 | ans-wild | Alberta | Central | PB2 |  | PA |  |  |  |  |  |
| A/mallard/Alberta/116/2007 | H3N8 | ans-wild | Alberta | Central | PB2 |  |  |  |  |  |  |  |
| A/mallard/Alberta/130/2007 | H3N8 | ans-wild | Alberta | Central | PB2 |  | PA |  |  |  |  |  |
| A/blue-wingedteal/Texas/Sg-00157/2007 | H4N6 | ans-wild | Texas | Central | PB2 |  | PA |  |  |  |  |  |
| A/mallard/California/8457/2008 | H6N2 | ans-wild | California | Pacific | PB2 |  | PA |  |  |  |  | NS |
| A/mallard/California/8834/2008 | H5N9 | ans-wild | California | Pacific | PB2 |  | PA |  |  |  |  |  |
| A/northernshoveler/California/9140/2008 | H1N9 | ans-wild | California | Pacific | PB2 |  | PA |  |  |  |  |  |
| A/northernshoveler/Washington/44249-645/2006 | H5N2 | ans-wild | Washington | Pacific | PB2 |  | PA |  |  |  |  |  |
| A/mallard/Alberta/76/2006 | H1N3 | ans-wild | Alberta | Central | PB2 |  | PA |  |  | NA |  |  |
| A/mallard/California/8212/2008 | H6N1 | ans-wild | California | Pacific | PB2 | PB1 | PA |  |  |  |  | NS |
| A/mallard/Alberta/254/2006 | H4N6 | ans-wild | Alberta | Central | PB2 |  | PA |  |  |  |  |  |
| A/mallard/Alberta/297/2006 | H4N6 | ans-wild | Alberta | Central | PB2 |  |  |  |  |  |  |  |
| A/bufflehead/California/HKWF205/2007 | H4N8 | ans-wild | California | Pacific | PB2 |  | PA |  |  |  |  |  |
| A/mallard/Alberta/121/2008 | H4N6 | ans-wild | Alberta | Central | PB2 |  | PA |  |  |  |  |  |
| A/mallard/Alberta/114/2007 | H4N6 | ans-wild | Alberta | Central | PB2 |  | PA |  |  |  |  |  |
| A/mallard/Alberta/160/2007 | H4N6 | ans-wild | Alberta | Central | PB2 |  | PA |  |  |  |  |  |
| A/northernshovelerMissouri/196/2009 | H10N3 | ans-wild | Missouri | Mississippi | PB2 | PB1 | PA |  |  | NA |  |  |
| A/northernpintail/InteriorAlaska/10BM02539R0/2010 | H7N3 | ans-wild | Alaska | Pacific | PB2 |  | PA | HA |  | NA |  |  |
| A/Americangreen-wingedteal/Wisconsin/10OS3127/2010 | H5N2 | ans-wild | Wisconsin | Mississippi | PB2 | PB1 | PA |  |  |  | M |  |
| A/green-wingedteal/California/K481/2006 | H1N3 | ans-wild | California | Pacific |  | PB1 |  |  |  |  |  |  |
| A/mallard/California/2556P/2011 | H3N8 | ans-wild | California | Pacific |  | PB1 |  |  |  |  |  |  |
| A/mallard/California/2556V/2011 | H3N8 | ans-wild | California | Pacific |  | PB1 |  |  |  |  |  |  |
| A/mallard/California/1390/2010 | H7N5 | ans-wild | California | Pacific |  | PB1 |  | HA | NP |  |  |  |
| A/mallard/Missouri/350/2009 | H11N9 | ans-wild | Missouri | Mississippi |  | PB1 |  |  |  |  |  |  |
| A/mallard/Missouri/10MO0391/2010 | H10N7 | ans-wild | Missouri | Mississippi |  | PB1 |  |  |  |  | M |  |
| A/mallard/Illinois/10OS3786/2010 | H10N7 | ans-wild | Illinois | Mississippi |  | PB1 |  |  |  |  | M | NS |
| A/mallard/Illinois/10OS4111/2010 | H10N7 | ans-wild | Illinois | Mississippi |  | PB1 |  |  |  |  | M |  |
| A/mallard/California/6471/2008 | H3N8 | ans-wild | California | Pacific |  | PB1 |  |  |  |  |  |  |
| A/ringneckedduck/California/HKWF402/2007 | H6N1 | ans-wild | California | Pacific |  | PB1 |  |  |  |  |  |  |
| A/northernshoveler/California/44363-062/2007 | H9N2 | ans-wild | California | Pacific |  | PB1 |  |  |  |  |  |  |
| A/blue-wingedteal/Ohio/566/2006 | H7N9 | ans-wild | Ohio | Mississippi |  | PB1 |  | HA |  |  |  |  |
| A/Americangreen-wingedteal/California/44287-373/2007 | H8N4 | ans-wild | California | Pacific |  | PB1 |  |  |  |  |  |  |
| A/commongoldeneye/Wisconsin/10OS4202/2010 | H7N6 | ans-wild | Wisconsin | Mississippi |  | PB1 |  | HA |  |  |  |  |
| A/blue-wingedteal/Texas/Sg-00074/2007 | H4N8 | ans-wild | Texas | Central |  | PB1 |  |  |  |  |  |  |
| A/green-wingedteal/California/123/2012 | H1N1 | ans-wild | California | Pacific |  | PB1 | PA |  |  |  |  |  |
| A/mallard/California/2589P/2011 | H4N1 | ans-wild | California | Pacific |  | PB1 |  |  |  |  |  |  |
| A/mallard/California/2533V/2011 | H4N6 | ans-wild | California | Pacific |  | PB1 |  |  |  |  |  |  |
| A/mallard/California/2555V/2011 | H11N9 | ans-wild | California | Pacific |  | PB1 |  |  |  |  |  |  |
| A/mallard/California/2563P/2011 | H4N6 | ans-wild | California | Pacific |  | PB1 |  |  |  |  |  |  |
| A/mallard/California/2595V/2011 | H12N5 | ans-wild | California | Pacific |  | PB1 |  |  |  |  |  |  |
| A/mallard/California/2536V/2011 | H5N1 | ans-wild | California | Pacific |  | PB1 |  |  |  |  |  |  |
| A/mallard/California/2527V/2011 | H5N1 | ans-wild | California | Pacific |  | PB1 |  |  |  |  |  |  |
| A/mallard/California/2531V/2011 | H5N1 | ans-wild | California | Pacific |  | PB1 |  |  | NP |  |  |  |
| A/mallard/California/2559P/2011 | H5N8 | ans-wild | California | Pacific |  | PB1 |  |  |  |  |  |  |
| A/Americangreen-wingedteal/InteriorAlaska/10BM05376R0/2010 | H3N8 | ans-wild | Alaska | Pacific |  | PB1 |  |  |  |  |  |  |
| A/northernshoveler/InteriorAlaska/10BM05491R0/2010 | H3N8 | ans-wild | Alaska | Pacific |  | PB1 |  |  |  |  |  |  |
| A/northernshoveler/InteriorAlaska/10BM05487R0/2010 | H3N8 | ans-wild | Alaska | Pacific |  | PB1 |  |  |  |  |  |  |
| A/northernshoveler/InteriorAlaska/10BM05382R0/2010 | H3N8 | ans-wild | Alaska | Pacific |  | PB1 |  |  |  |  |  |  |
| A/bufflehead/California/3118/2011 | H4N8 | ans-wild | California | Pacific |  | PB1 |  |  |  |  |  |  |
| A/mallard/California/2396/2010 | H5N2 | ans-wild | California | Pacific |  | PB1 |  |  |  |  |  |  |
| A/Americangreen-wingedteal/Illinois/10OS1598/2010 | H4N8 | ans-wild | Illinois | Mississippi |  | PB1 |  |  |  |  | M |  |
| A/mallard/Wisconsin/10OS3066/2010 | H6N2 | ans-wild | Wisconsin | Mississippi |  | PB1 |  |  |  |  |  |  |
| A/mallard/Ohio/1695/2009 | H4N6 | ans-wild | Ohio | Atlantic |  | PB1 |  |  |  |  |  |  |
| A/duck/InteriorAlaska/7MP1550/2007 | H4N6 | ans-wild | Alaska | Pacific |  | PB1 |  |  |  |  |  |  |
| A/mallard/InteriorAlaska/5/2007 | H3N8 | ans-wild | Alaska | Pacific |  | PB1 |  |  |  |  |  |  |
| A/northernshoveler/InteriorAlaska/2/2007 | H3N8 | ans-wild | Alaska | Pacific |  | PB1 |  |  |  |  |  |  |
| A/mallard/California/K752/2006 | H10N7 | ans-wild | California | Pacific |  | PB1 |  |  |  |  |  |  |
| A/northernshoveler/Mississippi/09OS025/2009 | H12N5 | ans-wild | Mississippi | Mississippi |  | PB1 |  |  |  |  |  |  |
| A/Americangreen-wingedteal/California/44363-002/2007 | H11N5 | ans-wild | California | Pacific |  | PB1 |  |  |  |  |  |  |
| A/mallard/Illinois/3974/2009 | H5N2 | ans-wild | Illinois | Mississippi |  | PB1 |  |  |  |  |  |  |
| A/blue-wingedteal/Wisconsin/10OS2862/2010 | H3N2 | ans-wild | Wisconsin | Mississippi |  | PB1 |  |  |  |  |  |  |
| A/woodduck/Wisconsin/10OS2778/2010 | H3N8 | ans-wild | Wisconsin | Mississippi |  | PB1 |  |  |  |  |  |  |
| A/mallard/Wisconsin/10OS2773/2010 | H3N8 | ans-wild | Wisconsin | Mississippi |  | PB1 |  |  |  |  |  |  |
| A/blue-wingedteal/Texas/Sg-00085/2007 | H3N6 | ans-wild | Texas | Central |  | PB1 |  |  |  |  |  |  |
| A/blue-wingedteal/Texas/Sg-00079/2007 | H3N8 | ans-wild | Texas | Central |  | PB1 |  |  |  |  |  |  |
| A/blue-wingedteal/Louisiana/Sg-00224/2007 | H3N8 | ans-wild | Louisiana | Mississippi |  | PB1 |  |  |  |  |  |  |
| A/blue-wingedteal/Texas/Sg-00188/2007 | H4N8 | ans-wild | Texas | Central |  | PB1 |  |  |  |  |  |  |
| A/mallard/Illinois/3747/2009 | H6N1 | ans-wild | Illinois | Mississippi |  | PB1 |  |  |  |  |  |  |
| A/bufflehead/Illinois/4016/2009 | H4N8 | ans-wild | Illinois | Mississippi |  | PB1 |  |  |  |  |  |  |
| A/mallard/Quebec/10969/2006 | H2N3 | ans-wild | Quebec | Atlantic |  | PB1 |  |  |  | NA |  |  |
| A/mallard/Quebec/16566/2005 | H11N2 | ans-wild | Quebec | Atlantic |  | PB1 |  |  |  |  |  |  |
| A/mallard/Quebec/11247/2006 | H3N2 | ans-wild | Quebec | Atlantic |  | PB1 |  |  |  |  |  |  |
| A/mallard/Quebec/11194/2006 | H3N2 | ans-wild | Quebec | Atlantic |  | PB1 |  |  |  |  |  |  |
| A/mallard/Quebec/11093/2006 | H3N2 | ans-wild | Quebec | Atlantic |  | PB1 |  |  |  |  |  |  |
| A/mallard/Illinois/08OS2315/2008 | H4N6 | ans-wild | Illinois | Mississippi |  | PB1 |  |  |  |  |  |  |
| A/blue-wingedteal/Wisconsin/2649/2009 | H6N1 | ans-wild | Wisconsin | Mississippi |  | PB1 |  |  | NP |  |  |  |
| A/mallard/California/8416/2008 | H6N1 | ans-wild | California | Pacific |  |  | PA |  |  |  |  |  |
| A/greaterwhite-frontedgoose/California/6548/2008 | H6N1 | ans-wild | California | Pacific |  |  | PA |  |  |  |  |  |
| A/Americanwigeon/California/9044/2008 | H6N1 | ans-wild | California | Pacific |  |  | PA |  |  |  |  |  |
| A/northernpintail/California/8470/2008 | H6N1 | ans-wild | California | Pacific |  |  | PA |  |  |  |  |  |
| A/gadwall/California/8708/2008 | H6N1 | ans-wild | California | Pacific |  |  | PA |  |  |  |  |  |
| A/green-wingedteal/MD/648/2004 | H7N3 | ans-wild | Maryland | Atlantic |  |  |  | HA |  |  |  |  |
| A/green-wingedteal/Ohio/648/2004 | H7N3 | ans-wild | Ohio | Mississippi |  |  |  | HA |  |  |  |  |
| A/blue-wingedteal/MD/658/2004 | H7N3 | ans-wild | Maryland | Atlantic |  |  |  | HA |  |  |  |  |
| A/blue-wingedteal/Ohio/658/2004 | H7N3 | ans-wild | Ohio | Mississippi |  |  |  | HA |  | NA |  |  |
| A/chicken/BritishColumbia/NS-01827-4/2004 | H7N3 | gal-domestic | BritishColumbia | Pacific |  |  |  | HA |  | NA |  |  |
| A/chicken/BritishColumbia/CN-7/2004 | H7N3 | gal-domestic | BritishColumbia | Pacific |  |  |  | HA |  |  |  |  |
| A/chicken/Canada/314514-1/2005 | H7N3 | gal-domestic | BritishColumbia | Pacific |  |  |  | HA |  |  |  |  |
| A/chicken/BritishColumbia/04 | H7N3 | gal-domestic | BritishColumbia | Pacific |  |  |  | HA |  | NA |  |  |
| A/blue-wingedteal/Texas/578575/2002 | H7N1 | ans-wild | Texas | Central |  |  |  | HA |  |  |  |  |
| A/blue-wingedteal/Texas/578585/2002 | H7N3 | ans-wild | Texas | Central |  |  |  | HA |  |  |  |  |
| A/mallard/Alberta/24/01 | H7N3 | ans-wild | Alberta | Central |  |  |  | HA |  |  |  |  |
| A/mallard/Alberta/34/2001 | H7N1 | ans-wild | Alberta | Central |  |  |  | HA |  |  |  |  |
| A/mallard/Alberta/22/2001 | H7N3 | ans-wild | Alberta | Central |  |  |  | HA |  |  |  |  |
| A/northernshoveler/California/HKWF1026/2007 | H7N3 | ans-wild | California | Pacific |  |  |  | HA |  | NA |  |  |
| A/green-wingedteal/California/11275/2008 | H7N3 | ans-wild | California | Pacific |  |  |  | HA |  | NA |  |  |
| A/green-wingedteal/California/1841/2009 | H7N3 | ans-wild | California | Pacific |  |  |  | HA |  |  |  |  |
| A/blackscoter/NewBrunswick/00014/2009 | H7N6 | ans-wild | NewBrunswick | Atlantic |  |  |  | HA |  |  |  |  |
| A/blackscoter/NewBrunswick/00003/2009 | H7N6 | ans-wild | NewBrunswick | Atlantic |  |  |  | HA |  |  |  |  |
| A/chicken/SK/HR-00011/2007 | H7N3 | gal-domestic | Saskatchewan | Central |  |  |  | HA |  | NA |  |  |
| A/mallard/InteriorAlaska/6MP0984/2006 | H7N3 | ans-wild | Alaska | Pacific |  |  |  | HA |  | NA |  | NS |
| A/cinnamonteal/California/JN1310/2007 | H7N3 | ans-wild | California | Pacific |  |  |  | HA |  | NA |  |  |
| A/mallard/Alberta/243/2006 | H7N3 | ans-wild | Alberta | Central |  |  |  | HA |  | NA |  |  |
| A/northernpintail/InteriorAlaska/8MP0262R2/2008 | H7N3 | ans-wild | Alaska | Pacific |  |  |  | HA |  | NA |  |  |
| A/mallard/Ohio/11OS2010/2011 | H7N8 | ans-wild | Ohio | Mississippi |  |  |  | HA |  |  |  |  |
| A/northernshoveler/Wisconsin/10OS3226/2010 | H7N3 | ans-wild | Wisconsin | Mississippi |  |  |  | HA |  | NA |  |  |
| A/mallard/Wisconsin/10OS3171/2010 | H7N3 | ans-wild | Wisconsin | Mississippi |  |  |  | HA |  | NA |  |  |
| A/mallard/InteriorAlaska/10CH00016R0/2010 | H7N3 | ans-wild | Alaska | Pacific |  |  |  | HA |  |  |  |  |
| A/northernpintail/InteriorAlaska/10BM09015R0/2010 | H7N6 | ans-wild | Alaska | Pacific |  |  |  | HA |  |  |  |  |
| A/mallard/InteriorAlaska/10BM04564R1/2010 | H7N3 | ans-wild | Alaska | Pacific |  |  |  | HA |  |  |  |  |
| A/mallard/InteriorAlaska/10BM10829R0/2010 | H7N3 | ans-wild | Alaska | Pacific |  |  |  | HA |  |  |  |  |
| A/mallard/InteriorAlaska/10BM09528R0/2010 | H7N3 | ans-wild | Alaska | Pacific |  |  |  | HA |  |  |  |  |
| A/northernpintail/InteriorAlaska/10BM10476R0/2010 | H7N3 | ans-wild | Alaska | Pacific |  |  |  | HA |  |  |  |  |
| A/mallard/InteriorAlaska/10BM09563R0/2010 | H7N3 | ans-wild | Alaska | Pacific |  |  |  | HA |  |  |  |  |
| A/northernpintail/InteriorAlaska/10BM10166R0/2010 | H7N3 | ans-wild | Alaska | Pacific |  |  |  | HA |  |  |  |  |
| A/northernpintail/InteriorAlaska/10BM11208R0/2010 | H7N3 | ans-wild | Alaska | Pacific |  |  |  | HA |  |  |  |  |
| A/northernpintail/InteriorAlaska/10BM07399R0/2010 | H7N3 | ans-wild | Alaska | Pacific |  |  |  | HA |  |  |  |  |
| A/northernpintail/InteriorAlaska/10BM07469R0/2010 | H7N3 | ans-wild | Alaska | Pacific |  |  |  | HA |  |  |  |  |
| A/mallard/InteriorAlaska/10BM07072R0/2010 | H7N3 | ans-wild | Alaska | Pacific |  |  |  | HA |  |  |  |  |
| A/mallard/InteriorAlaska/10BM12534R0/2010 | H7N3 | ans-wild | Alaska | Pacific |  |  |  | HA |  |  |  |  |
| A/mallard/InteriorAlaska/10BM07085R0/2010 | H7N3 | ans-wild | Alaska | Pacific |  |  |  | HA |  |  |  |  |
| A/northernpintail/InteriorAlaska/10BM06524R0/2010 | H7N3 | ans-wild | Alaska | Pacific |  |  |  | HA |  |  |  |  |
| A/gadwall/Missouri/10OS4731/2010 | H7N3 | ans-wild | Missouri | Mississippi |  |  |  | HA |  |  |  |  |
| A/northernshoveler/Mississippi/11OS289/2011 | H7N3 | ans-wild | Mississippi | Mississippi |  |  |  | HA |  |  |  |  |
| A/guineafowl/Nebraska/17096-1/2011 | H7N9 | gal-domestic | Nebraska | Central |  |  |  | HA |  |  |  |  |
| A/goose/Nebraska/17097-4/2011 | H7N9 | ans-wild | Nebraska | Central |  |  |  | HA |  |  |  |  |
| A/Americangreen-wingedteal/Illinois/10OS4014/2010 | H7N3 | ans-wild | Illinois | Mississippi |  |  |  | HA |  |  |  |  |
| A/Americangreen-wingedteal/Illinois/10OS3329/2010 | H7N7 | ans-wild | Illinois | Mississippi |  |  |  | HA | NP |  | M |  |
| A/northernshoveler/Mississippi/11OS202/2011 | H7N7 | ans-wild | Mississippi | Mississippi |  |  |  | HA |  |  |  |  |
| A/northernshoveler/Missouri/10OS4632/2010 | H7N7 | ans-wild | Missouri | Mississippi |  |  |  | HA |  |  |  |  |
| A/Americangreen-wingedteal/Mississippi/11OS250/2011 | H7N3 | ans-wild | Mississippi | Mississippi |  |  |  | HA |  |  |  |  |
| A/Americangreen-wingedteal/Mississippi/11OS255/2011 | H7N7 | ans-wild | Mississippi | Mississippi |  |  |  | HA |  |  |  |  |
| A/Americanblackduck/Wisconsin/10OS3949/2010 | H7N8 | ans-wild | Wisconsin | Mississippi |  |  |  | HA |  |  |  |  |
| A/northernshoveler/Missouri/10OS4750/2010 | H7N3 | ans-wild | Missouri | Mississippi |  |  |  | HA |  |  |  |  |
| A/northernshoverl/Mississippi/11OS145/2011 | H7N9 | ans-wild | Mississippi | Mississippi |  |  |  | HA |  |  |  |  |
| A/mallard/Missouri/10MO0551/2010 | H7N7 | ans-wild | Missouri | Mississippi |  |  |  | HA | NP |  |  |  |
| A/mallard/Missouri/220/2009 | H7N3 | ans-wild | Missouri | Mississippi |  |  |  | HA |  | NA |  |  |
| A/mallard/Missouri/10MO053/2010 | H7N4 | ans-wild | Missouri | Mississippi |  |  |  | HA |  |  | M |  |
| A/Americanblackduck/NewBrunswick/00344/2010 | H7N7 | ans-wild | NewBrunswick | Atlantic |  |  |  | HA | NP |  |  |  |
| A/green-wingedteal/NewBrunswick/00392/2010 | H7N7 | ans-wild | NewBrunswick | Atlantic |  |  |  | HA | NP |  |  |  |
| A/northernshoveler/California/JN1447/2007 | H7N2 | ans-wild | California | Pacific |  |  |  | HA |  |  |  |  |
| A/ruddyturnstone/DelawareBay/124/2007 | H7N3 | cha-wild | Delaware | Atlantic |  |  |  | HA |  |  |  |  |
| A/mallard/California/HKWF1971/2007 | H7N7 | ans-wild | California | Pacific |  |  |  | HA |  |  |  |  |
| A/greenwingedteal/California/AKS1370/2008 | H7N3 | ans-wild | California | Pacific |  |  |  | HA |  |  |  |  |
| A/mallard/NovaScotia/02286/2007 | H7N4 | ans-wild | NovaScotia | Atlantic |  |  |  | HA |  |  |  |  |
| A/Americanblackduck/NewBrunswick/04388/2007 | H7N3 | ans-wild | NewBrunswick | Atlantic |  |  |  | HA | NP | NA |  |  |
| A/Americanblackduck/NB/2538/2007 | H7N3 | ans-wild | NewBrunswick | Atlantic |  |  |  | HA |  | NA |  |  |
| A/Americanblackduck/NewBrunswick/02493/2007 | H7N3 | ans-wild | NewBrunswick | Atlantic |  |  |  | HA | NP | NA |  |  |
| A/Americanblackduck/NewBrunswick/02490/2007 | H7N3 | ans-wild | NewBrunswick | Atlantic |  |  |  | HA | NP |  |  |  |
| A/Canadagoose/BC/3752/2007 | H7N3 | ans-wild | BritishColumbia | Pacific |  |  |  | HA |  | NA |  |  |
| A/northernshoveler/California/28327/2007 | H7N3 | ans-wild | California | Pacific |  |  |  | HA |  | NA |  |  |
| A/northernshoveler/California/44287-364/2007 | H7N3 | ans-wild | California | Pacific |  |  |  | HA |  | NA |  |  |
| A/cinnamonteal/California/JN611/2006 | H7N3 | ans-wild | California | Pacific |  |  |  | HA |  |  |  |  |
| A/northernshoveler/California/27820/2007 | H7N3 | ans-wild | California | Pacific |  |  |  | HA |  |  |  |  |
| A/Americangreen-wingedteal/California/44287-084/2007 | H7N3 | ans-wild | California | Pacific |  |  |  | HA |  |  |  |  |
| A/Americangreen-wingedteal/California/44242-906/2007 | H7N3 | ans-wild | California | Pacific |  |  |  | HA |  | NA |  |  |
| A/northernshoveler/California/44287-164/2007 | H7N7 | ans-wild | California | Pacific |  |  |  | HA |  |  |  |  |
| A/Northernshoveler/NC/6412-052/2005 | H7N6 | ans-wild | NorthCarolina | Atlantic |  |  |  | HA |  |  |  |  |
| A/mallard/Delaware/418/2005 | H7N3 | ans-wild | Delaware | Atlantic |  |  |  | HA |  | NA |  |  |
| A/northernshoveler/NorthCarolina/6412-050/2005 | H7N3 | ans-wild | NorthCarolina | Atlantic |  |  |  | HA |  |  |  |  |
| A/northernshoveler/NorthCarolina/674-516/2005 | H7N3 | ans-wild | NorthCarolina | Atlantic |  |  |  | HA |  |  |  |  |
| A/Americangreen-wingedteal/California/28228/2007 | H7N6 | ans-wild | California | Pacific |  |  |  | HA |  |  |  |  |
| A/Americangreen-wingedteal/California/44287-305/2007 | H7N6 | ans-wild | California | Pacific |  |  |  | HA |  |  |  |  |
| A/northernshoveler/California/27985/2007 | H7N6 | ans-wild | California | Pacific |  |  |  | HA |  |  |  |  |
| A/northernshoveler/California/44287-179/2007 | H7N6 | ans-wild | California | Pacific |  |  |  | HA |  |  |  |  |
| A/northernshoveler/California/HKWF1372C/2007 | H7N3 | ans-wild | California | Pacific |  |  |  | HA |  | NA |  |  |
| A/ruddyturnstone/NewJersey/604/2006 | H7N4 | cha-wild | NewJersey | Atlantic |  |  |  | HA |  |  |  |  |
| A/ruddyturnstone/NewJersey/490/2006 | H7N3 | cha-wild | NewJersey | Atlantic |  |  |  | HA |  |  |  |  |
| A/ruddyturnstone/NewJersey/215/2006 | H7N3 | cha-wild | NewJersey | Atlantic |  |  |  | HA |  |  |  |  |
| A/sanderling/NewJersey/369/2006 | H7N3 | pas-wild | NewJersey | Atlantic |  |  |  | HA |  |  |  |  |
| A/ruddyturnstone/Delaware/752/2006 | H7N7 | cha-wild | Delaware | Atlantic |  |  |  | HA |  |  |  |  |
| A/ruddyturnstone/NewJersey/612/2006 | H7N3 | cha-wild | NewJersey | Atlantic |  |  |  | HA |  |  |  |  |
| A/sanderling/NewJersey/355/2006 | H7N3 | pas-wild | NewJersey | Atlantic |  |  |  | HA |  |  |  |  |
| A/laughinggull/DelawareBay/46/2006 | H7N3 | cha-wild | Delaware | Atlantic |  |  |  | HA |  | NA |  |  |
| A/laughinggull/DelawareBay/42/2006 | H7N3 | cha-wild | Delaware | Atlantic |  |  |  | HA |  | NA |  |  |
| A/ruddyturnstone/Delaware/890/2006 | H7N3 | cha-wild | Delaware | Atlantic |  |  |  | HA |  |  |  |  |
| A/ruddyturnstone/NewJersey/176/2006 | H7N3 | cha-wild | NewJersey | Atlantic |  |  |  | HA |  |  |  |  |
| A/laughinggull/DelawareBay/50/2006 | H7N3 | cha-wild | Delaware | Atlantic |  |  |  | HA |  |  |  |  |
| A/ruddyturnstone/NewJersey/204/2006 | H7N3 | cha-wild | NewJersey | Atlantic |  |  |  | HA |  |  |  |  |
| A/ruddyturnstone/DelawareBay/281/2006 | H7N3 | cha-wild | Delaware | Atlantic |  |  |  | HA |  |  |  |  |
| A/ruddyturnstone/DelawareBay/283/2006 | H7N3 | cha-wild | Delaware | Atlantic |  |  |  | HA |  |  |  |  |
| A/ruddyturnstone/DelawareBay/262/2006 | H7N3 | cha-wild | Delaware | Atlantic |  |  |  | HA |  | NA |  |  |
| A/ruddyturnstone/NewJersey/224/2006 | H7N3 | cha-wild | NewJersey | Atlantic |  |  |  | HA |  |  |  |  |
| A/ruddyturnstone/DelawareBay/290/2006 | H7N4 | cha-wild | Delaware | Atlantic |  |  |  | HA |  |  |  |  |
| A/ruddyturnstone/NewJersey/589/2006 | H7N3 | cha-wild | NewJersey | Atlantic |  |  |  | HA |  |  |  |  |
| A/ruddyturnstone/Delaware/892/2006 | H7N7 | cha-wild | Delaware | Atlantic |  |  |  | HA |  |  |  |  |
| A/ruddyturnstone/NewJersey/576/2006 | H7N3 | cha-wild | NewJersey | Atlantic |  |  |  | HA |  |  |  |  |
| A/laughinggull/DelawareBay/6/2006 | H7N3 | cha-wild | Delaware | Atlantic |  |  |  | HA |  | NA |  |  |
| A/shorebird/DelawareBay/513/2006 | H7N3 | cha-wild | Delaware | Atlantic |  |  |  | HA |  |  |  |  |
| A/ruddyturnstone/Delaware/789/2006 | H7N3 | cha-wild | Delaware | Atlantic |  |  |  | HA |  |  |  |  |
| A/ruddyturnstone/Delaware/779/2006 | H7N3 | cha-wild | Delaware | Atlantic |  |  |  | HA |  |  |  |  |
| A/shorebird/DelawareBay/332/2006 | H7N3 | cha-wild | Delaware | Atlantic |  |  |  | HA |  | NA |  |  |
| A/ruddyturnstone/NewJersey/562/2006 | H7N3 | cha-wild | NewJersey | Atlantic |  |  |  | HA |  |  |  |  |
| A/shorebird/Delaware/22/06 | H7N3 | cha-wild | Delaware | Atlantic |  |  |  | HA |  | NA |  |  |
| A/shorebird/DelawareBay/560/2006 | H7N3 | cha-wild | Delaware | Atlantic |  |  |  | HA |  | NA |  |  |
| A/avian/DelawareBay/226/2006 | H7N3 | cha-wild | Delaware | Atlantic |  |  |  | HA |  |  |  |  |
| A/redknot/NewJersey/AI06-096/2006 | H7N3 | cha-wild | NewJersey | Atlantic |  |  |  | HA |  |  |  |  |
| A/shorebird/DelawareBay/555/2006 | H7N3 | cha-wild | Delaware | Atlantic |  |  |  | HA |  |  |  |  |
| A/northernshoveler/Washington/44249-749/2006 | H7N3 | ans-wild | Washington | Pacific |  |  |  | HA |  | NA |  |  |
| A/mallard/Illinois/10OS3599/2010 | H6N1 | ans-wild | Illinois | Mississippi |  |  |  |  | NP |  |  |  |
| A/mallard/Louisiana/476670-4/2007 | H5N2 | ans-wild | Louisiana | Mississippi |  |  |  |  | NP |  |  |  |
| A/blue-wingedteal/Illinois/10OS2988/2010 | H4N6 | ans-wild | Illinois | Mississippi |  |  |  |  | NP |  | M |  |
| A/blue-wingedteal/Illinois/10OS1546/2010 | H3N6 | ans-wild | Illinois | Mississippi |  |  |  |  | NP |  | M |  |
| A/Americangreen-wingedteal/Wisconsin/2530/2009 | H6N2 | ans-wild | Wisconsin | Mississippi |  |  |  |  | NP |  |  |  |
| A/northernpintail/Alberta/8/2009 | H3N8 | ans-wild | Alberta | Central |  |  |  |  | NP |  |  |  |
| A/blue-wingedteal/NovaScotia/01009/2010 | H4N6 | ans-wild | NovaScotia | Atlantic |  |  |  |  | NP |  |  |  |
| A/mallard/California/5351/2009 | H1N1 | ans-wild | California | Pacific |  |  |  |  | NP |  |  |  |
| A/mallard/Ohio/11OS1992/2011 | H3N8 | ans-wild | Ohio | Mississippi |  |  |  |  | NP |  |  |  |
| A/mallard/Illinois/10OS3676/2010 | H3N8 | ans-wild | Illinois | Mississippi |  |  |  |  | NP |  |  |  |
| A/Americangreen-wingedteal/Wisconsin/2743/2009 | H1N1 | ans-wild | Wisconsin | Mississippi |  |  |  |  | NP |  |  |  |
| A/green-wingedteal/Minnesota/Sg-00131/2007 | H3N2 | ans-wild | Minnesota | Mississippi |  |  |  |  | NP |  |  |  |
| A/mallard/Ohio/1688/2009 | H12N5 | ans-wild | Ohio | Mississippi |  |  |  |  | NP |  |  |  |
| A/mallard/Wisconsin/2712/2009 | H3N6 | ans-wild | Wisconsin | Mississippi |  |  |  |  | NP |  |  |  |
| A/northernshoveler/Wisconsin/2508/2009 | H4N2 | ans-wild | Wisconsin | Mississippi |  |  |  |  | NP |  |  |  |
| A/mallard/Minnesota/Sg-00133/2007 | H4N6 | ans-wild | Minnesota | Mississippi |  |  |  |  | NP |  |  |  |
| A/mallard/Missouri/MO130/2005 | H11N3 | ans-wild | Missouri | Mississippi |  |  |  |  | NP | NA |  |  |
| A/wildbird/Minnesota/460613/2006 | H5N2 | ans-wild | Minnesota | Mississippi |  |  |  |  | NP |  |  |  |
| A/mallard/Wisconsin/08OS2271/2008 | H11N9 | ans-wild | Wisconsin | Mississippi |  |  |  |  | NP |  |  |  |
| A/mallard/Wisconsin/08OS2841/2008 | H2N3 | ans-wild | Wisconsin | Mississippi |  |  |  |  | NP | NA |  |  |
| A/greenwingedteal/Ohio/468160/2006 | H5N2 | ans-wild | Ohio | Mississippi |  |  |  |  | NP |  |  |  |
| A/blue-wingedteal/Wisconsin/3060/2009 | H3N2 | ans-wild | Wisconsin | Mississippi |  |  |  |  | NP |  |  |  |
| A/Americanblackduck/Wisconsin/2542/2009 | H4N2 | ans-wild | Wisconsin | Mississippi |  |  |  |  | NP |  |  |  |
| A/mallard/Wisconsin/3165/2009 | H1N1 | ans-wild | Wisconsin | Mississippi |  |  |  |  | NP |  |  |  |
| A/blue-wingedteal/Missouri/10MO013/2010 | H3N1 | ans-wild | Missouri | Mississippi |  |  |  |  | NP |  |  |  |
| A/mallard/California/2540P/2011 | H3N8 | ans-wild | California | Pacific |  |  |  |  | NP |  |  |  |
| A/mallard/California/2550V/2011 | H3N8 | ans-wild | California | Pacific |  |  |  |  | NP |  |  |  |
| A/mallard/California/2549V/2011 | H3N8 | ans-wild | California | Pacific |  |  |  |  | NP |  |  |  |
| A/northernshoveler/California/4020/2011 | H4N3 | ans-wild | California | Pacific |  |  |  |  | NP | NA |  |  |
| A/mallard/NovaScotia/00372/2010 | H7N7 | ans-wild | NovaScotia | Atlantic |  |  |  |  | NP |  |  |  |
| A/long-tailedduck/Wisconsin/10OS3915/2010 | H3N6 | ans-wild | Missouri | Mississippi |  |  |  |  | NP |  |  |  |
| A/mallard/Wisconsin/10OS3845/2010 | H5N2 | ans-wild | Wisconsin | Mississippi |  |  |  |  | NP |  |  |  |
| A/mallard/Wisconsin/2719/2009 | H4N2 | ans-wild | Wisconsin | Mississippi |  |  |  |  | NP |  |  |  |
| A/long-tailedduck/Wisconsin/10OS3919/2010 | H10N6 | ans-wild | Mississippi | Mississippi |  |  |  |  | NP |  |  |  |
| A/mallard/Mississippi/10OS4494/2010 | H1N1 | ans-wild | Mississippi | Mississippi |  |  |  |  | NP |  |  |  |
| A/mallard/Missouri/10MO0333/2010 | H11N9 | ans-wild | Missouri | Mississippi |  |  |  |  | NP |  |  |  |
| A/northernshoveler/California/3676/2010 | H8N2 | ans-wild | California | Pacific |  |  |  |  | NP |  |  |  |
| A/mallard/Wisconsin/10OS3144/2010 | H6N1 | ans-wild | Wisconsin | Mississippi |  |  |  |  | NP |  |  |  |
| A/northernshoveler/California/3483/2010 | H12N5 | ans-wild | California | Pacific |  |  |  |  | NP |  |  |  |
| A/Americangreen-wingedteal/Illinois/10OS3368/2010 | H7N7 | ans-wild | Illinois | Mississippi |  |  |  |  | NP |  | M |  |
| A/northernshoveler/Illinois/10OS3619/2010 | H11N9 | ans-wild | Illinois | Mississippi |  |  |  |  | NP |  |  |  |
| A/mallard/California/2590V/2011 | H4N6 | ans-wild | California | Pacific |  |  |  |  | NP |  |  |  |
| A/mallard/California/2578P/2011 | H3N8 | ans-wild | California | Pacific |  |  |  |  | NP |  |  |  |
| A/mallard/California/2566P/2011 | H4N8 | ans-wild | California | Pacific |  |  |  |  | NP |  |  |  |
| A/mallard/California/2555P/2011 | H11N9 | ans-wild | California | Pacific |  |  |  |  | NP |  |  |  |
| A/mallard/California/2563V/2011 | H4N6 | ans-wild | California | Pacific |  |  |  |  | NP |  |  |  |
| A/mallard/California/2595P/2011 | H12N5 | ans-wild | California | Pacific |  |  |  |  | NP |  |  |  |
| A/Americanwigeon/Iowa/10OS2748/2010 | H2N2 | ans-wild | Iowa | Mississippi |  |  |  |  | NP |  |  |  |
| A/mallard/Iowa/10OS2721/2010 | H2N2 | ans-wild | Iowa | Mississippi |  |  |  |  | NP |  |  |  |
| A/mallard/Illinois/10OS4179/2010 | H11N2 | ans-wild | Illinois | Mississippi |  |  |  |  | NP |  |  |  |
| A/Americangreen-wingedteal/Mississippi/11OS90/2011 | H11N9 | ans-wild | Mississippi | Mississippi |  |  |  |  | NP |  |  |  |
| A/mallard/Washington/44256-522/2006 | H11N3 | ans-wild | Washington | Pacific |  |  |  |  |  | NA |  |  |
| A/lesserscaup/Wisconsin/3964/2009 | H10N3 | ans-wild | Wisconsin | Mississippi |  |  |  |  |  | NA |  |  |
| A/mallard/Alberta/319/2009 | H2N3 | ans-wild | Alberta | Central |  |  |  |  |  | NA |  |  |
| A/mallard/Alberta/152/2006 | H1N3 | ans-wild | Alberta | Central |  |  |  |  |  | NA |  |  |
| A/mallard/Ohio/11OS1966/2011 | H10N3 | ans-wild | Ohio | Mississippi |  |  |  |  |  | NA |  |  |
| A/mallard/Missouri/10MO0550/2010 | H11N3 | ans-wild | Missouri | Mississippi |  |  |  |  |  | NA |  |  |
| A/mallard/Illinois/3051/2009 | H11N3 | ans-wild | Illinois | Mississippi |  |  |  |  |  | NA |  |  |
| A/mallard/Mississippi/11OS34/2011 | H1N3 | ans-wild | Mississippi | Mississippi |  |  |  |  |  | NA |  |  |
| A/mallard/California/1505/2010 | H4N3 | ans-wild | California | Pacific |  |  |  |  |  | NA |  |  |
| A/mallard/InteriorAlaska/10BM05347R0/2010 | H7N3 | ans-wild | Alaska | Pacific |  |  |  |  |  | NA |  |  |
| A/blue-wingedteal/Alberta/346/2007 | H4N3 | ans-wild | Alberta | Central |  |  |  |  |  | NA |  |  |
| A/northernshoveler/California/HKWF1005/2007 | H10N3 | ans-wild | California | Pacific |  |  |  |  |  | NA |  |  |
| A/northernshoveler/California/HKWF1370/2007 | H10N3 | ans-wild | California | Pacific |  |  |  |  |  | NA |  |  |
| A/mallard/Quebec/16334/2005 | H5N3 | ans-wild | Quebec | Atlantic |  |  |  |  |  | NA |  |  |
| A/mallard/Alberta/242/2004 | H2N3 | ans-wild | Alberta | Central |  |  |  |  |  | NA |  |  |
| A/mallard/Alberta/35/2009 | H2N3 | ans-wild | Alberta | Central |  |  |  |  |  | NA |  |  |
| A/mallard/Alberta/417/2009 | H2N3 | ans-wild | Alberta | Central |  |  |  |  |  | NA |  |  |
| A/ring-billedgull/Quebec/G066/2010 | H1N3 | cha-wild | Quebec | Atlantic |  |  |  |  |  | NA |  |  |
| A/ring-billedgull/Quebec/G139/2010 | H1N3 | cha-wild | Quebec | Atlantic |  |  |  |  |  | NA |  |  |
| A/northernpintail/SK/4612/2010 | H5N3 | ans-wild | Saskatchewan | Central |  |  |  |  |  | NA |  |  |
| A/northernpintail/SK/4628/2010 | H5N3 | ans-wild | Saskatchewan | Central |  |  |  |  |  | NA |  |  |
| A/ring-billedgull/Quebec/G018/2010 | H1N3 | cha-wild | Quebec | Atlantic |  |  |  |  |  | NA |  |  |
| A/mallard/Ohio/11OS2149/2011 | H2N3 | ans-wild | Ohio | Mississippi |  |  |  |  |  | NA |  |  |
| A/mallard/Wisconsin/08OS2844/2008 | H2N3 | ans-wild | Wisconsin | Mississippi |  |  |  |  |  | NA |  |  |
| A/mallard/Alberta/12017/2005 | H2N3 | ans-wild | Alberta | Central |  |  |  |  |  | NA |  |  |
| A/Americangreen-wingedteal/Illinois/10OS3343/2010 | H2N3 | ans-wild | Illinois | Mississippi |  |  |  |  |  | NA |  |  |
| A/northernshoveler/California/3046/2010 | H4N3 | ans-wild | California | Pacific |  |  |  |  |  | NA |  |  |
| A/northernshoveler/California/9781/2008 | H1N3 | ans-wild | California | Pacific |  |  |  |  |  | NA |  |  |
| A/ruddyturnstone/DelawareBay/108/2007 | H7N3 | cha-wild | Delaware | Atlantic |  |  |  |  |  | NA |  |  |
| A/northernshoveler/Mississippi/397/2010 | H1N3 | ans-wild | Mississippi | Mississippi |  |  |  |  |  | NA |  |  |
| A/mallard/California/1438/2010 | H2N3 | ans-wild | California | Pacific |  |  |  |  |  | NA |  |  |
| A/gadwall/California/44287-137/2007 | H5N3 | ans-wild | California | Pacific |  |  |  |  |  | NA |  |  |
| A/Americanwigeon/California/2930/2011 | H10N3 | ans-wild | California | Central |  |  |  |  |  | NA |  |  |
| A/Americancoot/Illinois/3405/2009 | H10N3 | gru-wild | Illinois | Mississippi |  |  |  |  |  | NA |  |  |
| A/Americancoot/Mississippi/09OS615/2009 | H10N3 | gru-wild | Mississippi | Pacific |  |  |  |  |  | NA |  |  |
| A/mallard/Alberta/80/2006 | H1N3 | ans-wild | Alberta | Central |  |  |  |  |  | NA |  |  |
| A/redknot/Delaware/441/2002 | H2N3 | cha-wild | Delaware | Atlantic |  |  |  |  |  | NA |  |  |
| A/shorebird/DelawareBay/53/2002 | H7N3 | cha-wild | Delaware | Atlantic |  |  |  |  |  | NA |  |  |
| A/blue-wingedteal/TX/2/01 | H7N3 | ans-wild | Texas | Central |  |  |  |  |  | NA |  |  |
| A/bluewingedteal/TX/75/2002 | H1N3 | ans-wild | Texas | Central |  |  |  |  |  | NA |  |  |
| A/bluewingedteal/TX/34/2002 | H1N3 | ans-wild | Texas | Central |  |  |  |  |  | NA |  |  |
| A/mallard/MN/479/2000 | H5N3 | ans-wild | Minnesota | Mississippi |  |  |  |  |  | NA |  |  |
| A/blackduck/Maryland/415/2001 | H7N3 | ans-wild | Maryland | Atlantic |  |  |  |  |  | NA |  |  |
| A/duck/PA/143585/2001 | H7N3 | ans-wild | Pennsylvania | Atlantic |  |  |  | HA |  | NA |  |  |
| A/mallard/Minnesota/Sg-00221/2007 | H10N3 | ans-wild | Minnesota | Mississippi |  |  |  |  |  | NA |  |  |
| A/Americancoot/California/20181-006/2007 | H10N3 | gru-wild | California | Pacific |  |  |  |  |  | NA |  |  |
| A/mallard/Alberta/254/2003 | H3N3 | ans-wild | Alberta | Central |  |  |  |  |  | NA |  |  |
| A/mallard/Quebec/11063/2006 | H2N3 | ans-wild | Quebec | Atlantic |  |  |  |  |  | NA |  | NS |
| A/mallard/Quebec/16485/2005 | H3N3 | ans-wild | Quebec | Atlantic |  |  |  |  |  | NA |  |  |
| A/northernshoveler/California/HKWF979/2007 | H3N3 | ans-wild | California | Pacific |  |  |  |  |  | NA |  |  |
| A/Americangreen-wingedteal/California/44287-713/2007 | H7N3 | ans-wild | California | Pacific |  |  |  |  |  | NA |  |  |
| A/cinnamonteal/California/44287-659/2007 | H10N3 | ans-wild | California | Pacific |  |  |  |  |  | NA |  |  |
| A/northernshoveler/California/JN770/2006 | H10N3 | ans-wild | California | Pacific |  |  |  |  |  | NA |  |  |
| A/northernshoveler/Washington/44249-603/2006 | H6N1 | ans-wild | Washington | Pacific |  |  |  |  |  |  | M |  |
| A/mallard/InteriorAlaska/10BM02980R0/2010 | H9N2 | ans-wild | Alaska | Pacific |  |  |  |  |  |  | M |  |
| A/northernpintail/InteriorAlaska/10BM14807R2/2010 | H9N2 | ans-wild | Alaska | Pacific |  |  |  |  |  |  | M |  |
| A/northernshoveler/InteriorAlaska/10BM16764R0/2010 | H9N2 | ans-wild | Alaska | Pacific |  |  |  |  |  |  | M |  |
| A/northernshoveler/California/9228/2008 | H4N6 | ans-wild | California | Pacific |  |  |  |  |  |  | M |  |
| A/northernshoveler/California/9267/2008 | H4N6 | ans-wild | California | Pacific |  |  |  |  |  |  | M |  |
| A/northernshoveler/California/10024/2008 | H4N4 | ans-wild | California | Pacific |  |  |  |  |  |  | M |  |
| A/mallard/Illinois/10OS4334/2010 | H10N7 | ans-wild | Illinois | Mississippi |  |  |  |  |  |  | M |  |
| A/blue-wingedteal/Illinois/10OS1563/2010 | H4N6 | ans-wild | Illinois | Mississippi |  |  |  |  |  |  | M |  |
| A/blue-wingedteal/Illinois/10OS1561/2010 | H4N6 | ans-wild | Illinois | Mississippi |  |  |  |  |  |  | M |  |
| A/mallard/Iowa/10OS2420/2010 | H4N6 | ans-wild | Iowa | Mississippi |  |  |  |  |  |  | M |  |
| A/mallard/Iowa/10OS2692/2010 | H4N2 | ans-wild | Iowa | Mississippi |  |  |  |  |  |  | M |  |
| A/Americanwigeon/California/HKWF42/2007 | H6N1 | ans-wild | California | Pacific |  |  |  |  |  |  | M |  |
| A/mallard/Missouri/10MO053/2010 | H7N4 | ans-wild | Missouri | Mississippi |  |  |  |  |  |  | M |  |
| A/mallard/Illinois/08OS2711/2008 | H10N7 | ans-wild | Illinois | Mississippi |  |  |  |  |  |  | M |  |
| A/mallard/Illinois/08OS2710/2008 | H10N7 | ans-wild | Illinois | Mississippi |  |  |  |  |  |  | M |  |
| A/Americangreen-wingedteal/Illinois/08OS2713/2008 | H10N7 | ans-wild | Illinois | Mississippi |  |  |  |  |  |  | M |  |
| A/mallard/Ohio/11OS2229/2011 | H5N2 | ans-wild | Ohio | Mississippi |  |  |  |  |  |  | M |  |
| A/mallard/Ohio/11OS2239/2011 | H5N2 | ans-wild | Ohio | Mississippi |  |  |  |  |  |  | M |  |
| A/mallard/Ohio/11OS2006/2011 | H5N2 | ans-wild | Ohio | Mississippi |  |  |  |  |  |  | M |  |
| A/mallard/Ohio/11OS1961/2011 | H5N2 | ans-wild | Ohio | Mississippi |  |  |  |  |  |  | M |  |
| A/mallard/Ohio/11OS2119/2011 | H5N2 | ans-wild | Ohio | Mississippi |  |  |  |  |  |  | M |  |
| A/mallard/Ohio/11OS2141/2011 | H3N2 | ans-wild | Ohio | Mississippi |  |  |  |  |  |  | M |  |
| A/mallard/Wisconsin/4230/2009 | H10N1 | ans-wild | Wisconsin | Mississippi |  |  |  |  |  |  | M |  |
| A/pintail/Alberta/84/2000 | H11N9 | ans-wild | Alberta | Central |  |  |  |  |  |  |  | NS |
| A/ruddyturnstone/DE/167/2004 | H10N7 | cha-wild | Delaware | Atlantic |  |  |  |  |  |  |  | NS |
| A/ruddyturnstone/NewJersey/110/2004 | H10N7 | cha-wild | NewJersey | Atlantic |  |  |  |  |  |  |  | NS |
| A/ruddyturnstone/NJ/238/2004 | H10N7 | cha-wild | NewJersey | Atlantic |  |  |  |  |  |  |  | NS |
| A/leastsandpiper/SouthCentralAlaska/1/2007 | H4N8 | cha-wild | Alaska | Pacific |  |  |  |  |  |  |  | NS |
| A/leastsandpiper/SouthCentralAlaska/7KW0434/2007 | H4N8 | cha-wild | Alaska | Pacific |  |  |  |  |  |  |  | NS |
| A/leastsandpiper/SouthCentralAlaska/2/2007 | H4N8 | cha-wild | Alaska | Pacific |  |  |  |  |  |  |  | NS |
| A/leastsandpiper/Alaska/7KW0411/2007 | H4N8 | cha-wild | Alaska | Pacific |  |  |  |  |  |  |  | NS |
| A/leastsandpiper/SouthCentralAlaska/3/2007 | H4N8 | cha-wild | Alaska | Pacific |  |  |  |  |  |  |  | NS |
| A/greenwingedteal/Ohio/464069/2006 | H5N2 | ans-wild | Ohio | Atlantic |  |  |  |  |  |  |  | NS |
| A/northernshoveler/Washington/44249-675/2006 | H10N2 | ans-wild | Washington | Pacific |  |  |  |  |  |  |  | NS |
| A/blue-wingedteal/Ohio/1339/2005 | H4N6 | ans-wild | Ohio | Mississippi |  |  |  |  |  |  |  | NS |
| A/green-wingedteal/California/K218/2005 | H4N6 | ans-wild | California | Pacific |  |  |  |  |  |  |  | NS |
| A/northernshoveler/Missouri/10OS4718/2010 | H10N7 | ans-wild | Missouri | Mississippi |  |  |  |  |  |  |  | NS |
| A/northernpintail/InteriorAlaska/6MP0792/2006 | H2N3 | ans-wild | Alaska | Pacific |  |  |  |  |  |  |  | NS |
| A/mallard/InteriorAlaska/6MP0038BR2/2006 | H2N3 | ans-wild | Alaska | Pacific |  |  |  |  |  |  |  | NS |
| A/mallard/InteriorAlaska/7MP0709/2007 | H3N8 | ans-wild | Alaska | Pacific |  |  |  |  |  |  |  | NS |
| A/northernshoveler/Alaska/7MP1113/2007 | H4N6 | ans-wild | Alaska | Pacific |  |  |  |  |  |  |  | NS |
| A/northernshoveler/InteriorAlaska/7MP1649/2007 | H3N8 | ans-wild | Alaska | Pacific |  |  |  |  |  |  |  | NS |
| A/northernshoveler/InteriorAlaska/7MP0953/2007 | H3N8 | ans-wild | Alaska | Pacific |  |  |  |  |  |  |  | NS |
| A/northernshoveler/InteriorAlaska/7MP0944/2007 | H3N8 | ans-wild | Alaska | Pacific |  |  |  |  |  |  |  | NS |
| A/northernpintail/InteriorAlaska/1/2007 | H3N8 | ans-wild | Alaska | Pacific |  |  |  |  |  |  |  | NS |
| A/ring-neckedduck/California/K90/2005 | H6N8 | ans-wild | California | Pacific |  |  |  |  |  |  |  | NS |
| A/mallard/Maryland/182/2006 | H5N2 | ans-wild | Maryland | Atlantic |  |  |  |  |  |  |  | NS |
| A/mallard/Quebec/11281/2006 | H2N3 | ans-wild | Quebec | Atlantic |  |  |  |  |  |  |  | NS |
| A/wildbird/Minnesota/460613-12/2006 | H5N2 | ans-wild | Minnesota | Mississippi |  |  |  |  |  |  |  | NS |
| A/greenwingedteal/Delaware/458672-5/2006 | H5N2 | ans-wild | Delaware | Atlantic |  |  |  |  |  |  |  | NS |
| A/mallard/California/19524-001/2005 | H6N8 | ans-wild | California | Pacific |  |  |  |  |  |  |  | NS |
| A/mallard/Alberta/330/2007 | H4N6 | ans-wild | Alberta | Central |  |  |  |  |  |  |  | NS |
| A/northernpintail/Alberta/265/2007 | H4N6 | ans-wild | Alberta | Central |  |  |  |  |  |  |  | NS |
| A/mallard/California/6469/2008 | H6N2 | ans-wild | California | Pacific |  |  |  |  |  |  |  | NS |
| A/pintailduck/Alberta/49/2003 | H12N5 | ans-wild | Alberta | Central |  |  |  |  |  |  |  | NS |
| A/mallard/Alberta/221/2006 | H12N6 | ans-wild | Alberta | Central |  |  |  |  |  |  |  | NS |
